# Bioengineered intestinal muscularis complexes with long-term spontaneous and periodic contractions

**DOI:** 10.1371/journal.pone.0195315

**Published:** 2018-05-02

**Authors:** Qianqian Wang, Ke Wang, R. Sergio Solorzano-Vargas, Po-Yu Lin, Christopher M. Walthers, Anne-Laure Thomas, Martín G. Martín, James C. Y. Dunn

**Affiliations:** 1 Department of Bioengineering, Henry Samueli School of Engineering and Applied Science, University of California Los Angeles, Los Angeles, California, United States of America; 2 Division of Pediatric Surgery, Department of Surgery, Stanford University School of Medicine, Stanford, California, United States of America; 3 Department of Computer Science, University of North Carolina at Chapel Hill, Chapel Hill, North Carolina, United States of America; 4 Department of Pediatrics, Division of Gastroenterology and Nutrition, Mattel Children’s Hospital and the David Geffen School of Medicine at UCLA, University of California Los Angeles, Los Angeles, California, United States of America; 5 Department of Bioengineering, Stanford University, Stanford, California, United States of America; Lewis Katz School of Medicine at Temple University, UNITED STATES

## Abstract

Although critical for studies of gut motility and intestinal regeneration, the *in vitro* culture of intestinal muscularis with peristaltic function remains a significant challenge. Periodic contractions of intestinal muscularis result from the coordinated activity of smooth muscle cells (SMC), the enteric nervous system (ENS), and interstitial cells of Cajal (ICC). Reproducing this activity requires the preservation of all these cells in one system. Here we report the first serum-free culture methodology that consistently maintains spontaneous and periodic contractions of murine and human intestinal muscularis cells for months. In this system, SMC expressed the mature marker myosin heavy chain, and multipolar/dipolar ICC, uniaxonal/multipolar neurons and glial cells were present. Furthermore, drugs affecting neural signals, ICC or SMC altered the contractions. Combining this method with scaffolds, contracting cell sheets were formed with organized architecture. With the addition of intestinal epithelial cells, this platform enabled up to 11 types of cells from mucosa, muscularis and serosa to coexist and epithelial cells were stretched by the contracting muscularis cells. The method constitutes a powerful tool for mechanistic studies of gut motility disorders and the functional regeneration of the engineered intestine.

## Introduction

In the small intestine, the mucosa processes partially digested food and absorbs nutrients while the muscularis actuates the peristaltic flow to transport luminal content aborally. Gut motility is central to its digestive and absorptive function. The intestinal muscularis contains various types of cells. Of these, smooth muscle cells, the enteric nervous system (ENS)[1,2], and the pacemaker interstitial cells of Cajal (ICC)[3] are three important players involved in the development of gut motility. Recent studies of intestinal tissue engineering have highlighted the importance of regenerating the functional intestinal muscularis[4–10]. A variety of systems derived from different cell sources, including pluripotent stem cells (PSC)[4–6], embryonic stem cells[7] and primary cells[8,9], have been established to accomplish this goal and different contractile activities were developed in these systems. Notably, spontaneous contractions have been generated in culture systems that contained both ICC and smooth muscle cells[4,6,11–14]. In addition, electrical-induced neurogenic contractions were also successfully produced[4,5,8] when ENS was introduced into culture. In one of the most recent studies, after *in vivo* incubation, both spontaneous contractions and electrical-induced neurogenic contractions were developed in a PSC-based culture system[4].

All of these approaches have substantially advanced the study of intestinal diseases and intestinal regeneration, yet contractions similar to those observed in native tissue have not been generated in previous *in vitro* culture systems. Freshly isolated intestinal muscle strips can display continuous, spontaneous and periodic contractions with distinct physical movements[15,16] (n = 21 animals, **S1 Video**). In this study, we sought to reproduce this type of contractions in cell culture by developing a serum-free culture methodology for intestinal muscularis cells (IMC).

IMC are cells isolated from the intestinal smooth muscle layers. For disease modeling and therapeutic testing of gut motility disorders, IMC are the most accessible cell source and the best representative of the various cellular components of the whole tissue. However, the applications of IMC are significantly limited by the technical difficulties in their long-term *in vitro* preservation. In the traditional serum-containing medium[8,14,17] (hereafter “**serum medium**”) for IMC culture, smooth muscle cells rapidly de-differentiate and lose their contractility[18–20] while ICC[12] and ENS[14] do not survive in long term. The most common and already commercialized methods to re-differentiate smooth muscle cells are to reduce the amount of serum and to add heparin in culture[21,22]. However, media developed through those methods are designed only for smooth muscle cell monoculture and lack essential nutrients for other cells including neurons and ICC in IMC. Various protocols have been developed to specifically culture primary smooth muscle cells[23], ICC[12,13], or enteric neural elements[24]—or two of the three[25] in combination—but here, for the first time, we developed a single culture medium (termed “**muscularis medium**”) that preserves all three in one system for an extended period and restored the spontaneous, rhythmic contractile function of IMC.

In this study, we first demonstrated from multiple aspects the significant enhancements of murine IMC culture in the new muscularis medium. Using video microscopy and contraction frequency analysis, we showed that in the muscularis medium, primary murine IMC, for the first time *in vitro*, exhibited continuous self-paced periodic contractions for over two months. Using immunofluorescence and RT-PCR analysis, we demonstrated that smooth muscle cells, ICC, neurons and glia, all thrived in the muscularis medium. In addition, smooth muscle cells expressed the mature marker myosin heavy chain while ICC, neurons and glial cells not only preserved a substantial morphological diversity but also formed self-organized networks. To further investigate whether different cells all functionally participated in the observed contractions, we used a collection of drugs targeting different cells and monitored the subsequent alternations of the contractile activity. As a result, we showed that functional smooth muscle cells in the muscularis medium responded to carbachol stimulation for contraction as well as to nitric oxide for relaxation. ICC preserved the pacemaker activity, which was indicated by the presence of the spontaneous calcium oscillations in the muscularis medium. Blocking the pacemaker activity of ICC by niflumic acid abolished the contractions of smooth muscle cells. Moreover, neuromuscular communication, especially the nitrergic neuromuscular inhibitory mechanism, was explored by TTX, DMPP, hexamethonium and L-NAME. In the second part of this study, the new medium was used to complement alternative techniques for demonstrating its potential applications in many fundamental and applied contexts. For example, with a 3D electrospun poly-caprolactone scaffold incorporated into our new culture system, a contracting IMC sheet was successfully regenerated. In combination with the culturing method of epithelial crypts, the muscularis medium not only supported the epithelium-IMC co-culture but also retained the spontaneous periodic contractions of IMC, providing a promising platform for studying the intestinal epithelium-muscularis interrelationship. Finally, a new medium for human IMC was formulated on the basis of the muscularis medium for murine IMC, which successfully maintained the contractility and the protein and gene expressions of human fetal IMC.

## Materials and methods

### Mice and human specimens

Wild-type mice (C57BL/6, Charles River Laboratories, Wilmington, MA) or mice expressing green fluorescent protein (GFP, C57BL/6-Tg(Actb-EGFP)1Osb/J, Jackson Laboratory, Bar Harbor, ME) were used in the study. Intestinal muscularis was isolated from 3 to 7-day-old mice. Intestinal crypts were isolated from 6-week-old mice. All animal studies were approved by Animal Research Committee and Office of Animal Research Oversight at University of California Los Angeles (UCLA) as protocol number 2005–169 or by Stanford University Institutional Animal Care and Use Committee A3213-01. All efforts were made to minimize animal pain and suffering. For human studies, de-identified healthy small intestine tissues from discarded surgical samples of infant, teenager or adult patients were obtained through the Department of Pathology Translational Pathology Core Laboratory at UCLA. Fourteen to 18-week-old fetal bowels were obtained upon the written informed consent from each patient. All human studies were approved by UCLA Institutional Review Board.

### IMC isolation

IMC isolation was performed using previously published protocols[14]. Intestines were removed and placed on ice in Hank’s Buffered Saline Solution lacking magnesium and calcium (HBSS, Life Technologies, Carlsbad, CA) with 1x antibiotic-antimycotic (ABAM, Invitrogen, Carlsbad, CA). Intestinal muscles containing both longitudinal and circular muscle layers were carefully stripped off from the intestine using forceps, collected in HBSS buffer with 1x ABAM in 15 ml conical tubes on ice, and centrifuged at 1,000 rpm for 5 minutes. Next, muscles were re-suspended into the freshly-made digestion buffer: 1 mg/ml collagenase from Clostridium histolyticum, Type XI (Sigma, St. Louis, MO) in HBSS. The content was gently mixed by taping the bottom of the tubes for 10 times, then incubated at 37°C for 30 minutes. During digestion, at each 10-min interval, the content was re-mixed by tapping the bottom of the conical tube for 3–5 times. 10 ml DMEM, low glucose, GlutaMAX™ Supplement, pyruvate (Life Technologies, Carlsbad, CA) with 10% fetal bovine serum (FBS, Invitrogen) and 1x ABAM was added to terminate the process. The cells were pelleted by centrifugation at 1,000 rpm for 5 minutes and re-suspended in the serum medium prior to culture. IMC isolation from the human tissue followed the same methods used for mice.

### Cell culture

Three different culture media were used in this study: 1. Serum medium: DMEM with 10% FBS and 1x ABAM; 2. The muscularis medium: Advanced DMEM/Ham’s F-12 (Invitrogen) with 1x N2 (Invitrogen), 1x B27 (Invitrogen), 1 mM N-acetylcysteine (Nac, Sigma), 10 mM HEPES (Invitrogen), 2 mM GlutaMAX (Invitrogen), 100 ng/mL recombinant murine Noggin (Stemgent, Cambridge, MA), 1 μg/mL recombinant human R-spondin1 (R&D Systems, Inc., Minneapolis, MN), 10 μM Y27632 (Peprotech, Rocky Hill, NJ) and ABAM; and 3. The human muscularis medium: subtracting Nac from the muscularis medium. For murine IMC, IMC in the serum medium were plated onto the 24-well culture plates (Corning, Corning, NY) with a density of 320,000 cells/well (or 48-well plates at 160,000 cells/well). IMC in all the conditions were cultured in a 37°C incubator with 10% carbon dioxide. Media were changed every other day. For culture using (human) muscularis media, IMC were first pre-cultured in the serum medium for 2 days to allow cells to attach and grow, then transferred to (human) muscularis media. For the (human) muscularis media, all the components can be pre-mixed and stored for two weeks at 4°C, except noggin, R-spondin1 and Y27632, which need to be added right before changing media. IMC were passaged by TrypLE™ Select Enzyme (Life technologies). A 28 gauge syringe (Fisher) was used to break up the cell clusters. The passaged cells were plated with the serum medium. For cells cultured in totally serum-free conditions, 10% FBS in the serum medium in the pre-incubation was replaced by 10% (10 g/100 ml) bovine serum albumin, Fraction V (BSA, Fisher Scientific, Pittsburgh, PA) and cells were pre-incubated for 4 days instead of 2 days. In most cases, IMC were unfiltered. For the experiment testing whether or not the muscularis medium was effective on filtered IMC, IMC were filtered by a 70 micron nylon filter (Corning, Corning, NY)[14].

### Isolation of intestinal crypts

Murine intestinal crypts were isolated by a previously reported method[26]. Murine intestinal tissue was removed from the animal and cut open in cold phosphate buffered saline (PBS, Life Technologies). With mucosa surface facing up, the excess mucoid material was scrapped by the tweezer tips. Next the specimen was washed several times until the solution remained clear. The specimen then was cut into approximately 1 cm^2^ pieces, transferred into 30 ml of 2.5 mmol/L EDTA in PBS and incubated for 30 minutes at 4°C. At the end of incubation, 15 ml supernatant was discarded with intestinal fragments settled at the bottom of the tube. 15 ml of cold PBS was added into the tissue and the total 30 ml solution with tissue was vortexed for 3 seconds x 10 times. After the fragments settled down at the bottom, the supernatant was collected and saved on ice (Crypt fraction 1). 15 ml of PBS was added into the tissue again and the process was repeated six times (Crypt fraction 1–6). Samples then were centrifuged at 100 rcf for 2 minutes. About 13 ml of the supernatant was discarded and the pellets were re-suspended in the rest of the solution with the addition of 10% FBS. The purity of crypt fractions was examined under microscope. Several fractions were pooled together based on the need of experiments. The pooled sample was purified by the combination of a 100-μm and a 70-μm filter (BD Biosciences, Bedford, MA). Next the crypts were spun at 100 rcf for 2 minutes and resuspended at a density of 300 crypts in 25 μl Matrigel (BD Biosciences). The crypts with Matrigel were placed onto the 48-well culture plates. Matrigel was allowed to polymerize at 37°C for 15 minutes. Isolation of human crypts was conducted in a similar way except that instead of 2.5 mM EDTA, 16 mM EDTA with 1 mM Dithiothreitol was used in this procedure. Murine epithelium at Passage 0 to 1 and human epithelium from adult patients at Passage 11 to 12 were used for co-culture.

### Intestinal epithelium and IMC co-culture

IMC were cultured in the muscularis medium for 21 days before adding epithelial cells. For passaged epithelial cells, epithelial cells/Matrigel with culture media were removed from the wells and collected into Eppendorf tubes. The cells/Matrigel were quickly spun for 3 seconds x 3 times. Upon removal of the supernatant, 500 μl TrypLE™ Select Enzyme was added to digest the Matrigel for 5 minutes at 37°C. After the digestion, 500 μl DMEM with 10% FBS was added to each tube. The content in each tube was well mixed, and quickly spun for 3 seconds x 3 times. The supernatant was discarded. The pellet was resuspened into the muscularis medium (murine cells) or human muscularis medium (human cells) and directly placed onto the cultured IMC. For fresh crypts, crypts were resuspended into the muscularis medium (murine cells) and directly placed onto the cultured IMC after isolation. For each well on a 24-well plate, about 500 units of epithelial structures or crypts were seeded on IMC. Co-culture was maintained in a 37°C incubator with 5% carbon dioxide for 4 days.

### Contractile assessment

Contractions of IMC were analyzed using video microscopy. IMC formed contracting cell clusters when cultured in the (human) muscularis media. Fluorescence (for GFP^+^ IMC) and phase contrast (for non-GFP IMC) videos of the cultured cell clusters or fresh muscle strips were recorded by a camera connected to the Olympus IX71 or IX73 microscope with CellSens software (Olympus, Center Valley, PA) at room temperature (22°C to 25°C). Each video was acquired at 40x magnification which captured an area of about 3.7 mm^2^ for >30 seconds. Every periodically contracting cell cluster captured in videos was analyzed. Based on our previous work[14], custom MATLAB programs (**S1–S3** **Codes**) were written to estimate the frequency of contractions. We first manually selected the regions of interest (ROIs) on the contracting cell clusters. The contraction-relaxation cycles of the cells caused a periodical change of intensity in the selected ROIs (**S1 Fig**). For GFP^+^ cells, in the contracted state, the size of the cluster became smaller while the number of cells in each cluster remained the same, leading to an increase of the cell density, and subsequently fluorescently brighter cell clusters, i.e. an increase of the fluorescence intensity in ROIs (**S1A Fig**). When cells were relaxed, the size of cell clusters extended, the density of the cells decreased, and the clusters became dim, i.e. a decrease of the fluorescence intensity in ROIs (**S1A Fig**). If cells were not contracting, no obvious intensity change could be detected. For non-GFP cells, contractions were recorded under phase contrast mode (black and white). In contrast to GFP^+^ cells, non-GFP cell clusters in contraction state demonstrated a more compact and darker formulation than that in relaxation state, leading to a decrease in intensity (darker image, **S1B Fig**). In some rare cases, ROIs were selected at the periphery of contracting clusters and intensity in these ROIs changed when the cell clusters contracted to reveal the background underneath (**S1C Fig**). We hypothesized that the frequency of the intensity change can represent the frequency of the contractions. Using custom MATLAB programs, we measured the frequency of the intensity change. The averaged intensity value within ROIs for each frame was calculated and compared to that from the first frame in each stack to generate a normalized intensity profile. A Fast Fourier Transform (FFT) was performed on the average intensities for each ROI in the temporal domain. Contraction frequency was then identified as the frequency response with the second largest magnitude, the period of contractions as the reciprocal of the identified frequency. To acquire the sufficient sensitivity and a good signal/noise ratio for detecting the differences of the intensity among each frame, histogram equalization was used to suppress image noises and eliminate environmental illuminations prior to the FFT. For epithelium-IMC co-culture and human IMC culture, frames that captured the maximum contraction state and maximum relaxation state of the cell clusters were manually selected and extracted from the videos. Then, an optical-flow analysis was conducted based on previous work[27] to estimate and visualize the displacement of each pixel on the cell clusters between these two states.

### Intracellular Ca^2+^ imaging

IMC were cultured in the muscularis medium for 28 days. Then calcium flux was visualized using the Fluo-4 Direct™ Calcium Assay Kit[14,28] (Thermo Fisher Scientific) following the product protocol. Fluorescence intensity change caused by intracellular Ca^2+^ flux was recorded using video microscopy at room temperature (22°C to 25°C) and ROIs were selected. The frequency of fluorescence intensity change within the ROIs was quantified using a custom MATLAB script (**S2 Code**).

### Immunofluorescence

Immunostaining was performed following the typical protocol. Samples were fixed by formalin (Fisher Scientific) for 25 min at room temperature, permeabilized with 0.5% Triton X-100 and incubated in a blocking solution of 4% goat serum (Vector Laboratories, Burlingame, CA) with 2% BSA in PBS for 1 hour at room temperature. The primary antibodies were incubated overnight at 4°C, rinsed, and incubated with fluorescently-conjugated secondary antibodies for 2 hours at room temperature. All the antibodies were diluted into the blocking solution. The antibodies used were listed in **S1 Table**. For staining of c-Kit, IMC were cultured on the glass chamber slides (Fisher Scientific, seeding density: 250,000 cells/chamber), fixed by acetone (Fisher Scientific) for 30 min at 4°C and permeabilized with 0.1% Triton X-100 in blocking solution. Images were taken by the Olympus IX71 or IX73 microscope with CellSens software. Confocal images were taken by Inverted Zeiss LSM 880 Laser Scanning Confocal Microscope (Zeiss, Oberkochen, Germany) at Stanford Cell Science Imaging Facility.

### Quantitative real-time RT-PCR

RNA was isolated from cultured IMC, freshly isolated muscle strips or crypts (as the control) with a Qiashredder (Qiagen, Germantown, MD) and RNeasy kit (Qiagen). Quantitative real-time RT-PCR was carried out with QuantiTect Probe RT-PCR kit (Qiagen) on the 7500 Real Time PCR System (Applied Biosystems, Invitrogen). Relative expression was calculated based on the ΔΔCt method with *Gapdh* as reference. For human markers, *MYH11*, *C-KIT*, *TUBB3*, *GFAP* and *GAPDH*, real-time RT-PCR was performed with qScript™ One-Step SYBR® Green qRT-PCR Kit (Quanta Biosciences, Beverly, MA). Validated primers and probes used here were listed in **S1 Table**.

### Pharmacological responses

Prior to the tests, IMC were cultured in muscularis medium for 28–35 days. Carbachol (Thermo Fisher Scientific), sodium nitroprusside (SNP, Sigma), tetrodotoxin citrate (Tocris, Bristol, United Kingdom), 1,1-dimethyl-4-phenyl-piperazinium iodide (DMPP, Sigma), hexamethonium chloride (Sigma) and Nω-nitro-l-arginine methylester HCl (L-NAME, Sigma) were dissolved in distilled water as stock solution. Niflumic acid (Sigma) were dissolved in dimethyl sulphoxide (DMSO, ATCC, Manassas, VA). All stock solutions were prepared on the day of experimentation. Dilutions, pre-incubated to 37°C, were directly administrated into the bath medium. Each concentration for all drugs was non-cumulatively applied to individual samples. The doses and incubation times of each drug were chosen based on previous work in literature[4,14,16,29–38]. Carbachol and DMPP were applied while recording videos since the effects of carbachol and DMPP were immediate[14,31]. IMC with SNP, TTX and niflumic acid were incubated for 3 mins[29], 5 mins[4] and 15 mins[30] (respectively) at 37°C to obtain stable responses and videos were taken immediately after the incubation to record any change of cell contractility. For the study of neuronal nicotinic acetylcholine receptors, DMPP, or DMPP simultaneously with hexamethonium[38] was applied to the culture. For the examination of the nitrergic pathway in the neuromuscular coupling, samples were pretreated with L-NAME for 3 min at 37°C before the DMPP stimulation. Prior to the experiments, distilled water or DMSO alone was administrated into the culture in exactly the same way that we used to administrate the drugs (using the same volumes (≤1% of the bath medium), same incubation times, same mixing methods, etc.) and resulted in no obvious effects on the contraction frequency of IMC (**S2 Fig**, n = 3 bio-independent samples for each condition). For calculating the IC50 value of SNP, an area that contained about 10 cell clusters for each sample was recorded before and after the 3-min application of SNP. For each cell cluster present in each video, we counted the number of contractions of the same clusters within one minute (COM) before (set as control) and after the administration of SNP. The inhibition effect on the contraction frequency was expressed as percent decrease of COM from control. All the contractile assessments were conducted at room temperature (22 to 25°C).

### Electrospun scaffold

11% (w/w) Poly-caprolactone (PCL, Durect Lactel, Cupertino, CA) in 1,1,1,3,3,3-Hexafluoro-2-propanol (HFIP, Sigma) was prepared 1-day prior to electrospinning and well mixed. A customized electrospinning set was built in the lab with a syringe pump, a high voltage supplier and a rotating mandrel as the collector. The mandrel was 3 mm in diameter. The rotating speed was 3000 rpm. The experiment was conducted at 13.5 kV and the target volume for each scaffold was 0.15 mL. The scaffold was removed from the mandrel and cut into the size of a well of a 48-well plate. Scaffolds were coated by the neutralized collagen (Advanced BioMatrix, Carlsbad, CA) prior to cell seeding. The seeding density on each scaffold was 1 million per scaffold.

### Statistics

All the results were present as mean values ± standard deviations with n indicating the number of biologically independent samples. Differences between groups were evaluated using one-way analysis of variance (ANOVA) and Tukey’s post hoc method of multiple comparisons. For two-group comparison, tests for data variance were first performed. The two-tailed unpaired Student’s t-test was used for two groups with equal variances. The unpaired Student’s t-test with Welch’s correction was used for groups with unequal variances. Frequency counts were conducted in Origin Pro 2015 (Student Version, OriginLab Corp, Northampton, MA) for histograms showing the distribution of contraction periods at each time point. Differences between distributions were determined by the two-sided Kolmogorov–Smirnov test. A *p*-value <0.05 was treated as statistically significant. Based on the concentration-inhibition curve, the IC_50_ value of SNP was obtained by fitting the data to the sigmoidal dose–response model. All the statistical studies were carried out using OriginPro 2015 or 2016. Graphs were drawn using GraphPad Prism 6 (GraphPad Software Inc., San Diego CA).

## Results

### The development of the muscularis medium

We tested several medium formulations used to culture other muscle cells and also examined the medium used for intestinal epithelial cell culture[26,39–41] (EC medium) for the development of co-culture platforms. Interestingly, we found that EC medium supported the culture of IMC, with the appearance of the neural network but without spontaneous contractions (**S3 Fig**). We hypothesized that the EC medium could be modified into a new formulation suitable for IMC culture. We systematically removed one or more components of the EC medium, assessed the resultant effects on cultured IMC contractility and discovered that epidermal growth factor (EGF, a well-known stimulator of cell growth) in the EC medium prevented the IMC regular contractions (**S2 and S3 Tables**, **S1 Note**). Upon removal of EGF, cultured IMC displayed striking spontaneous contractility (**S2 and S3 Tables**, **S1 Note**, **S2 Video**). In contrast to the **serum medium**[8,14,17] for IMC culture, this new **muscularis medium** does not contain serum and has defined molecules added, including B27, N2, N-acetylcysteine, Noggin, R-spondin1, and Y27632.

### Long-term spontaneous and periodic contractions of murine IMC

**Fig 1:**
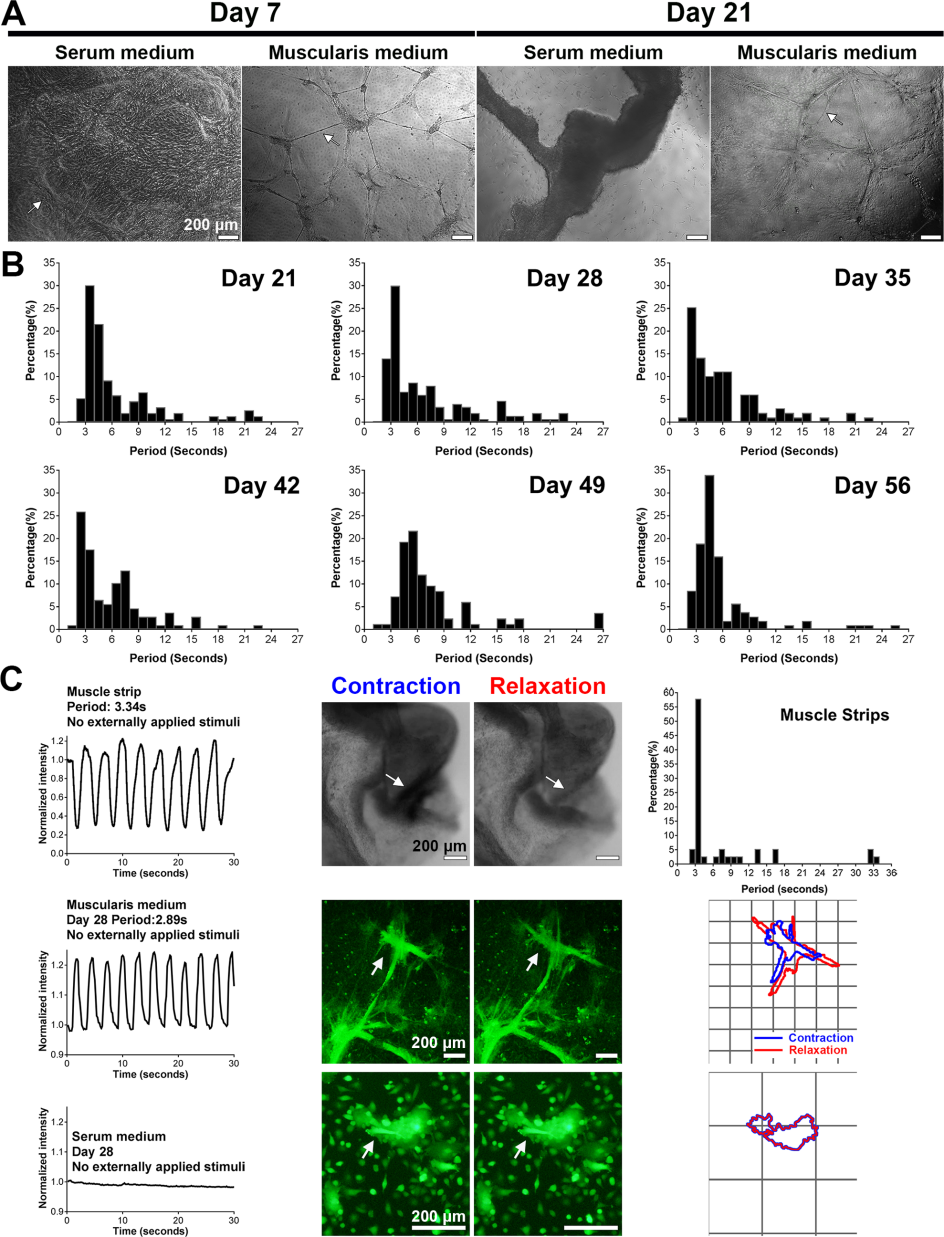
IMC in the muscularis medium exhibit long-term periodic and spontaneous contractions (no stimulation). (**A**) Representative phase contrast images of IMC in serum and muscularis media at day 7 and 21. Arrow in the first image points to the hill-and-valley pattern. Arrow in the second image indicates neurite-like connections between clusters. Arrow in the last image points to partially detached cell clusters. (**B**) Distributions of contraction periods of IMC in the muscularis medium at 21 (*153*, **4**; N = *153* cell clusters from n = **4** biologically independent samples), 28 (*173*, **6**), 35 (*99*, **3**), 42 (*108*, **3**), 49 (*83*, **3**) and 56 (*106*, **3**) days. No externally applied stimuli. See distributions for day 7 and 14 in **S4 Fig**. (**C**) Typical recordings of spontaneous periodic contractions (left), shape changes (arrow, middle) and outlines (right; grid, 200 μm) of the IMC clusters in the muscularis medium and the serum medium at day 28 as well as the recording (left) and shape changes (arrow, middle) of the contracting spot on the muscle strip. For the outline image of IMC in the serum medium: the blue line is thicker than the red line. Top right image shows the distribution of contraction periods of muscle strips (N = 38 spots from n = 9 animals). Scale bars in (**A**) and (**C**), 200 μm. All the contractile assessments were conducted at room temperature (22 to 25°C).

The muscularis medium potently supported the spontaneous periodic contractions of IMC (n = 80 bio-independent samples). Murine IMC formed interconnected cell clusters in the muscularis medium, a morphology different from that observed in the traditional serum medium (**Fig 1A**). Without any externally applied stimuli, most clusters initiated visible spontaneous contractions within 7 days, as indicated by the distinct change of the clusters’ physical sizes under microscope (**S3 Video**). The contraction was a coordinated activity of a group of cells, indicating the possible involvement of gap junction coupling (**S2** and S**3 Videos**). By day 21, contractions were faster and more regular than contractions at day 7 (**Fig 1B**, **S4 Fig**, **S2**and S**3 Videos**). Specifically, at day 28, the distribution of contraction periods of IMC clusters was not significantly different than that of fresh muscle strips (Kolmogorov–Smirnov test, *p*>0.05, **Fig 1B and 1C**, **S1–S3 Videos**). The contractions of IMC clusters resembled those of native tissue and persisted for at least 56 days (n = 4 bio-independent samples), with contraction periods clustering around 2–5 seconds (>50%, **Fig 1B and 1C**, **S1**–S3 **Videos**). We further observed that passaged IMC in the muscularis medium also generated similar contractions (**S4 Video**, n = 3 bio-independent samples). The muscularis medium was always effective, whether or not IMC were filtered prior to seeding (**S5 Video**, n = 3 bio-independent samples). In contrast, cells in the serum medium remained static (**Fig 1C**, **S6 Video**, n = 3 bio-independent samples).

### Maintenance of mature smooth muscle cells, ICC, neurons and glia

**Fig 2:**
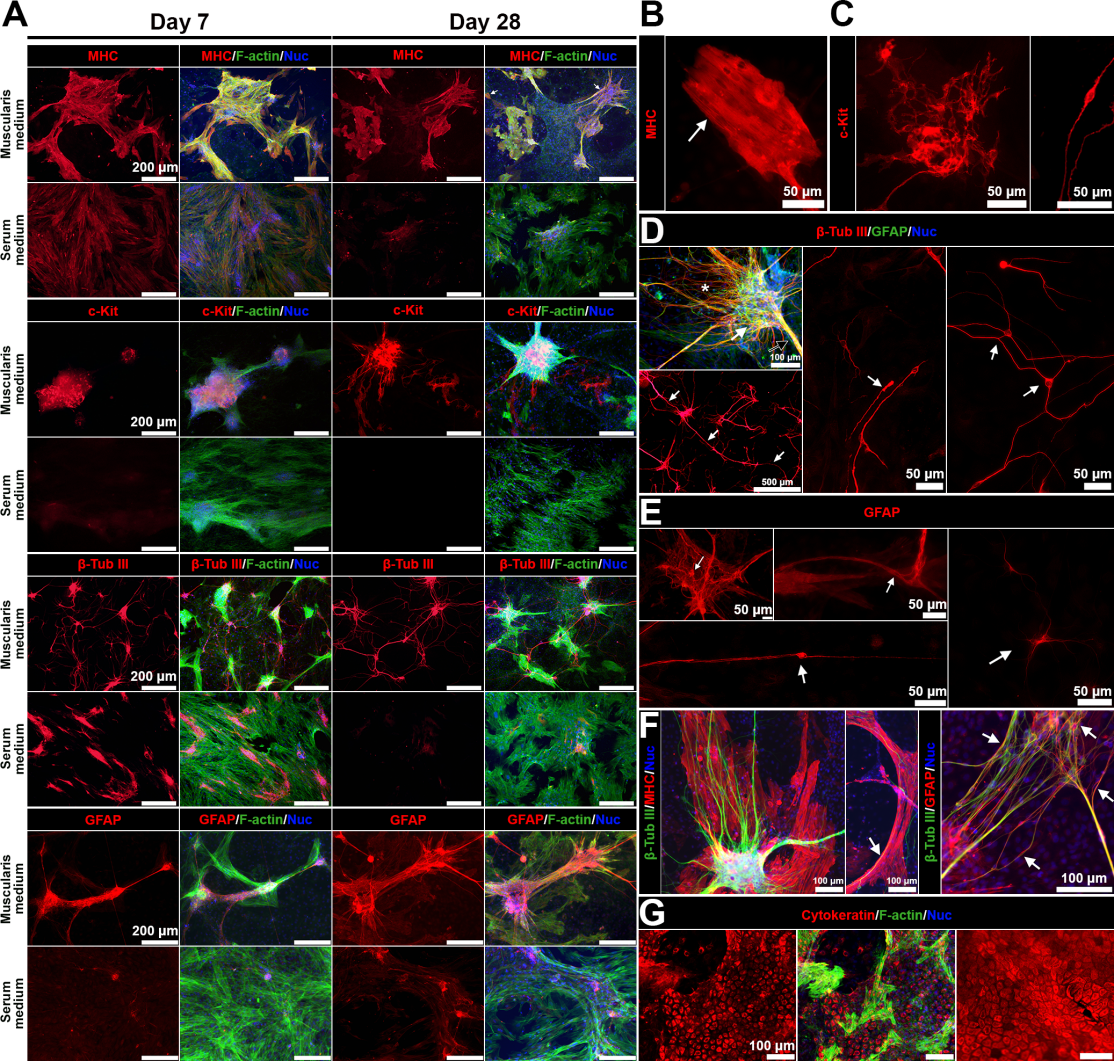
The muscularis medium maintains mature smooth muscle cells, ICC, neurons and glial cells. (**A**) Immunofluorescence of MHC, c-Kit, β-tubulin III, and GFAP in the serum or muscularis media at d7 and d28. Nuclei (DAPI, blue); F-actin (phalloidin, green). Scale bars, 200 μm. (**B**-**E**) Details of cells in the muscularis medium at day 28. (**B**) Filament bundles in contractile smooth muscle cells (arrow). (**C**) Multipolar ICC network (left) and dipolar ICC (right, day 21). (**D**) Ganglia-like neural aggregates (white arrow), thick neurite bundles (black arrow) and neural fibers (white asterisk) are present in the muscularis medium, top left, scale bar, 100 μm; neurites extend over 2,000 μm (left bottom, arrows, scale bars, 500 μm); and different types of neurons (middle and right). (**E**) Four different types of glial cells (arrows). (**F**) Close associations of smooth muscle cells, neurons and glial cells (arrows). (**G**) Serosal mesothelial cells (cytokeratin, red) in the muscularis medium at day 28 (left, middle) and in muscle strips (right). Scale bars, 100 μm.

Mature smooth muscle cells, ICC, neurons and glia all thrived in the muscularis medium as shown by immunofluorescence (n = 3 bio-independent samples). The protein marker myosin heavy chain (MHC) is expressed only when smooth muscle cells are mature[19,20]. Smooth muscle cells in the muscularis medium showed intense expression of MHC and displayed features associated with the mature phenotype, such as the typical fusiform shape and bundled microfilaments, indicating they were maintained at a differentiated, contractile state (**Fig 2A and 2B**).

The muscularis medium also effectively sustained ICC (c-Kit^+^[3]) which demonstrated different morphologies. Some of the c-Kit^+^ cells were dipolar, a morphology reminiscent of the shape of intramuscular ICC; some of the c-Kit^+^ cells were multipolar, similar to the morphology of myenteric ICC[42] (**Fig 2A and 2C**). Multipolar c-Kit^+^ cells connected to each other and formed networks (**Fig 2A and 2C**).

The immunofluorescence of β-tubulin III[43]and GFAP[24,44] demonstrated that IMC in the muscularis medium contained numerous neurons and glial cells (respectively). Together these cells reconstituted key morphological features of ENS[1,42], including ganglia-like neural aggregates, thick connective nerve strands out from these neural aggregates, and individual nerve fibers probably innervating smooth muscle cells (**Fig 2A and 2D–2F**). Again, neurons and glial cells with different morphologies were observed in the muscularis medium. We observed both uniaxonal neurons (similar to Dogiel type I morphology) and multipolar neurons (similar to Dogiel type II morphology) (**Fig 2D**). In addition, we were able to pinpoint four morphologically distinct subsets of glial cells[2] (**Fig 2E**).

Different cell types were closely associated with each other (**Fig 2F**) in the muscularis medium. The neural aggregates, ICC networks, and mature smooth muscle cells together formed periodically contracting intestinal muscularis complexes among the sheet of serosal mesothelial cells (**Fig 2F and 2G**). Over 2,000-μm-long neurites (**Fig 2D**), along with processes from glial cells, built large networks to connect these contracting intestinal muscularis complexes.

Compared with the muscularis medium, expressions of MHC, c-Kit, and GFAP were either low or totally absent in the traditional serum medium (**Fig 2A**). The expression of β-tubulin III existed at the early time point in the serum medium but dramatically decreased with time (**Fig 2A**).

**Fig 3:**
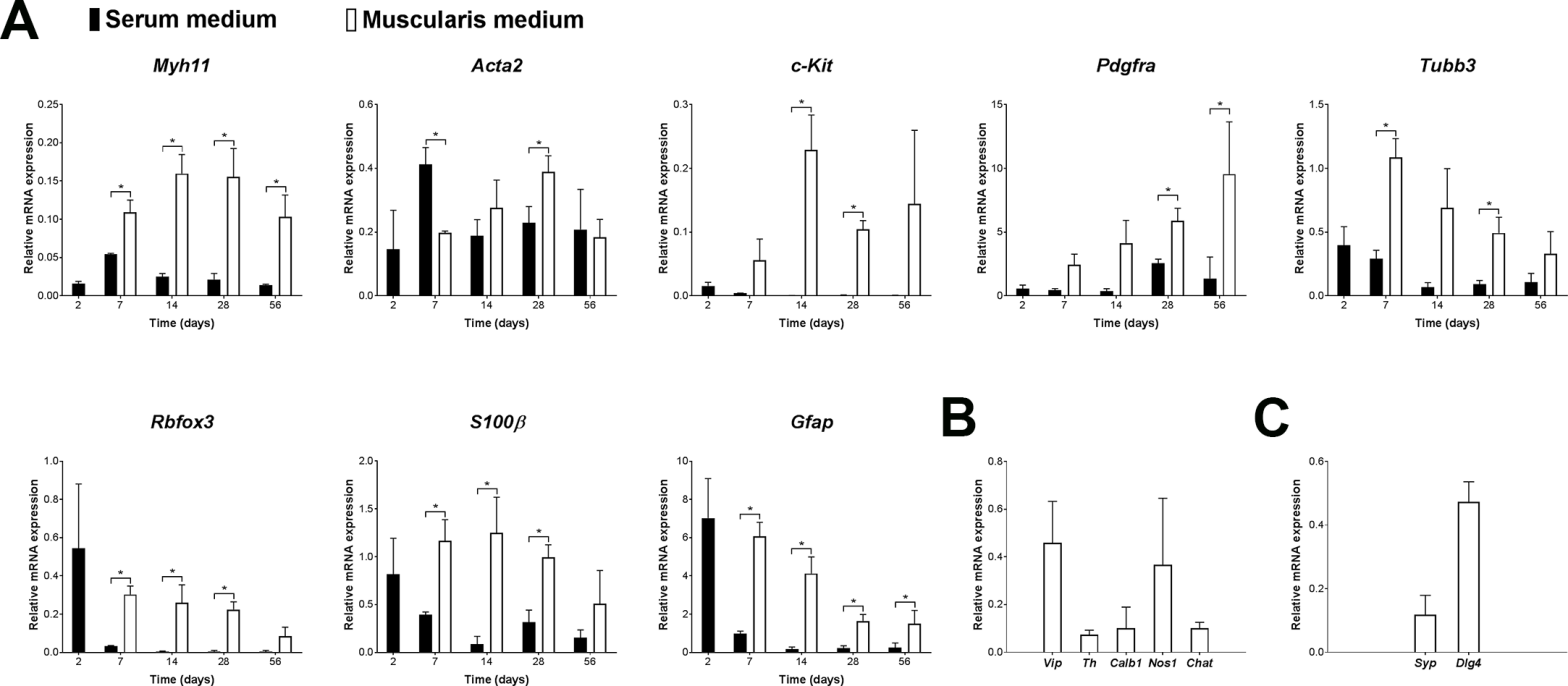
The muscularis medium maintains various cell types at the gene level. (**A**) Relative mRNA expression of indicated markers in serum and muscularis media at day 2 (pre-incubation in the serum medium), 7, 14, 28 and 56, real-time RT-PCR. (**B**) Relative mRNA expression of various enteric neuronal markers in the muscularis medium at day 28, real-time RT-PCR. (**C**) Relative mRNA expression of synaptogenesis-related markers in the muscularis medium at day 28, real-time RT-PCR. Control: muscle strips; housekeeping gene: *Gapdh*. Error bars, S.D. (n = 3 (**A**-**B**) or 4 (**C**) biologically independent samples). Two-tailed Student’s t-test, *p < 0.05.

The gene expression patterns examined by quantitative real-time RT-PCR further supported the presence of these various cell types. During the two-month culture, IMC in the muscularis medium had consistently higher gene expression of mature smooth muscle cells (*Myh11*), ICC (*c-Kit*), neurons (*Tubb3*, *Rbfox3*) and glial cells (*S100β*, *Gfap*) than in traditional serum medium (**Fig 3A**, n = 3 bio-independent samples). In both muscularis and serum media, cultured IMC maintained α-smooth muscle actin (*Acta2*), a marker that appears in both mature and synthetic smooth muscle cell phenotypes[22] (**Fig 3A**, n = 3 bio-independent samples). In addition, the platelet-derived growth factor receptor alpha-positive (PDGFRα^+^) cell is another important cell type fundamental to the pacemaker activities in the intestine[3]. IMC in the muscularis medium expressed high level of *Pdgfra*, suggesting the successful preservation of PDGFRα^+^ cells in the muscularis medium (**Fig 3A**, n = 3 bio-independent samples). Furthermore, different enteric neuronal markers (*Vip*, *Th*, *Calb1*, *Chat*, *Nos1*, **Fig 3B**, n = 3 bio-independent samples) were detected in the muscularis medium, indicating notable neuronal diversity in the system. IMC in the muscularis medium also expressed genes related to synaptogenesis, such as *Syp* (synaptophysin, a presynaptic marker[5]) and *Dlg4* (PSD-95, a postsynaptic marker[45]) (**Fig 3C**, n = 4 bio-independent samples).

### Role of ICC, neural networks and muscle in contractile activity

**Fig 4:**
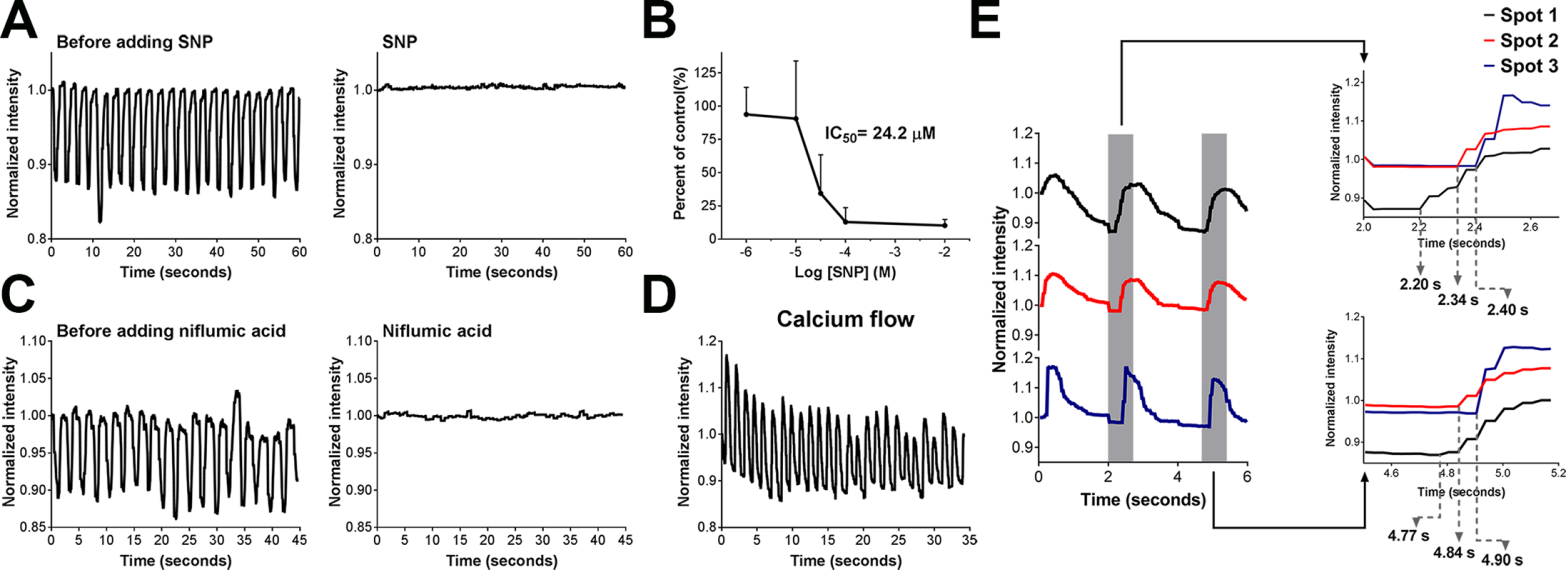
Role of smooth muscle cells and ICC in the observed contractions in the muscularis medium. (**A**) Representative recordings of the effect of 100 μM SNP on IMC cultured in the muscularis medium at d28 (**S9 Video**). (**B**) Concentration–response curve for frequency of the contractions in response to SNP. Error bars, S.D. (N = 42, 55, 36, 30, 42 cell clusters for -2, -4, -4.5, -5, -6 log [SNP] (M) respectively, each from n = 3 biologically independent samples). (**C**) Representative recordings of the effect of 300 μM niflumic acid on IMC cultured in the muscularis medium at d35 (**S10 Video**, n = 3 biologically independent samples). (**D**) Representative recordings of the spontaneous periodic Ca^2+^ oscillations in the muscularis medium at d28 (**S12 Video**, n = 3 biologically independent samples). (**E**) Ca^2+^ influx onset was propagated along correlated contracting spots (the first three contractions shown in **S13 Video**, n = 3 biologically independent samples). All the contractile assessments were conducted at room temperature (22 to 25°C).

To ascertain whether the neural network, ICC and smooth muscle cells contributed to the contractions observed in the muscularis medium, several drugs targeting each were tested in culture. The contractions were altered accordingly when smooth muscle cells were affected by adding carbachol or sodium nitroprusside (SNP), suggesting the involvement of functional smooth muscle cells in the contractile activity. We tested the effects of carbachol, a cholinergic agonist, at concentrations of 10 and 50 μM (usually from 0.1 to 100 μM[32–34] for studies of murine intestinal smooth muscle). Similar to previous observations[14,31,46,47], the addition of carbachol caused a tonic contraction (> 1 minute (50 μM), **S7 Video** (first a short version for a quick view of the drug effect, then the full version), for each concentration, n = 3 bio-independent samples). The effects of carbachol on IMC were similar to its action on muscle strips (**S8 Video**, for each concentration, n = 3 bio-independent samples). In contrast to carbachol, the smooth muscle relaxant SNP, a nitric oxide (NO) donor[31,48], reduced the frequency of the contractions in a dose-dependent manner with an IC_50_ value of 24 μM (**Fig 4A and 4B**, **S9 Video**, for each concentration, n = 3 bio-independent samples). Consistent with previous studies[4,16,31], at 100 μM, about 80–100% of the contractions were abolished by SNP (**Fig 4A**, **S9 Video**, n = 3 bio-independent samples).

The Ca^2+^-activated Cl^-^ channel, Anoctamin 1 (ANO 1), is essential to the pacemaker activity of ICC[30]. To determine whether the periodic contractions in the muscularis medium were ICC-dependent, we blocked ANO 1 channel by niflumic acid (300 μM[30], a concentration effective for murine intestine), which resulted in the inhibition of IMC contractions (**Fig 4C**, **S10** (IMC)-**S11** (muscle strips) **Videos**, n = 3 (IMC) or 6 (muscle strips) bio-independent samples).

In addition, smooth muscle contractions result from intracellular Ca^2+^ oscillations[49]. A functional ICC network produces periodic Ca^2+^ pulses to effect the contractile pattern[49]. To further examine the participation of ICC in the observed contractions, we loaded a fluorescent Ca^2+^ indicator into cultured IMC to visualize the intracellular Ca^2+^. Fluorescence intensity change caused by intracellular Ca^2+^ flux was recorded and quantified using a customized MATLAB script. The highest fluorescence intensity represented the highest Ca^2+^ level. We observed spontaneous and periodic Ca^2+^ oscillations of the contracting cell clusters in the muscularis medium (**Fig 4D**, **S12**and S**13 Videos**, n = 3 bio-independent samples). During contraction, the physical movements of cells followed the influx of Ca^2+^ with a short delay (0.03–0.27 seconds, **S12 Video**). The Ca^2+^ flux also propagated from one part of the cultured IMC to another. Correlated contracting clusters experienced the influx of Ca^2+^ one by one (**Fig 4E**, **S13 Video**). These results support the role of ICC in the spontaneous contractions of IMC in the muscularis medium.

**Fig 5:**
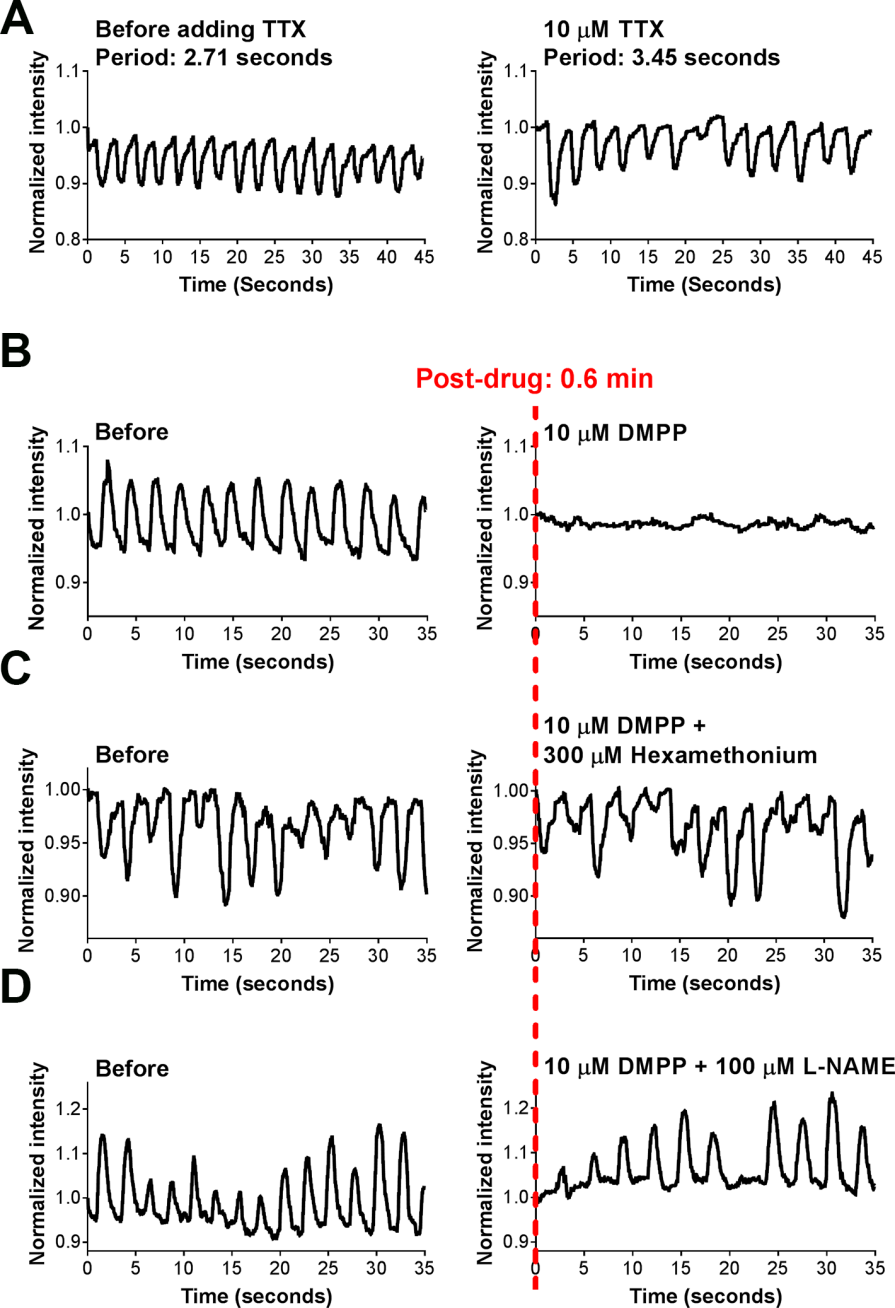
Role of the neural signals in the observed contractile activities in the muscularis medium. (**A**) Representative recordings of the effect of 10 μM TTX on IMC cultured in the muscularis medium at d28 (**S14**and S**15 Videos**). See **S5 Fig** for TTX effects on IMC at 400 μM and its effect on muscle strips at 10 μM, 400 μM and 1 mM. (**B**) Representative recordings of the effects of 10 μM DMPP on IMC cultured in the muscularis medium at d28 (**S16 Video**, n = 6 biologically independent samples). (**C**) Representative recordings of the effects of 10 μM DMPP simultaneously with 300 μM hexamethonium on IMC cultured in the muscularis medium at d28 (**S16 Video**, n = 3 biologically independent samples). (**D**) Representative recordings of the effects of 10 μM DMPP with 100 μM L-NAME (3 min pretreatment) on IMC cultured in the muscularis medium at d28 (**S16 Video**, n = 3 biologically independent samples). All the contractile assessments were conducted at room temperature (22 to 25°C).

Next, to investigate the neural signals in IMC culture, we applied the neural blocker tetrodotoxin (TTX) to IMC in the muscularis medium and also explored the nitrergic neuromuscular inhibitory mechanism for muscle relaxation. It has been shown that TTX ≤ 10 μM cannot block the ICC-involved spontaneous contractions[16,35]. Consistent with the literature, we observed that IMC in the muscularis medium and fresh muscle strips continued to contract after the administration of 10 μM TTX, but the frequency of the contractions was slightly altered (**Fig 5A**, **S5 Fig**, **S14 Video** (IMC)-**S15 Video** (muscle strips), each n = 6 bio-independent samples). TTX terminated the contractions of cultured IMC at 400 μM and severely disrupted the contractions of fresh muscle strips at 1 mM (**S5 Fig**, **S14**and S**15 Videos**, each n = 6 bio-independent samples).

In native tissue, stimulation of the nicotinic acetylcholine receptors (nAChR) on post-ganglionic nerves can activate inhibitory motor neurons to release NO, which is the major inhibitory neurotransmitter to cause muscularis relaxation[31,37,50]. In this study, we stimulated the neurons by a typical ganglionic nAChR agonist, 1,1-dimethyl-4-phenyl-piperazinium iodide (DMPP, 10 μM[4,31,36]), which elicited an immediate relaxation in the muscularis medium (**Fig 5B**, **S16 Video**, n = 6 bio-independent samples). Hexamethonium (300 μM[38]), as a ganglionic nAChR antagonist, inhibited the effect of DMPP (**Fig 5C**, **S16 Video**, n = 3 bio-independent samples). To further confirm the participation of NO in the muscle relaxation, we used Nω-nitro-l-arginine methylester HCl (L-NAME) to block the NO synthesis[16]. The relaxation evoked by DMPP was significantly attenuated in the presence of L-NAME (100 μM[16], **Fig 5D**, **S16 Video**, n = 3 bio-independent samples). These results together indicate the involvement of NO-dependent neurogenic activities in the relaxation of IMC in the muscularis medium.

### Periodically contracting IMC sheets with scaffolds

**Fig 6:**
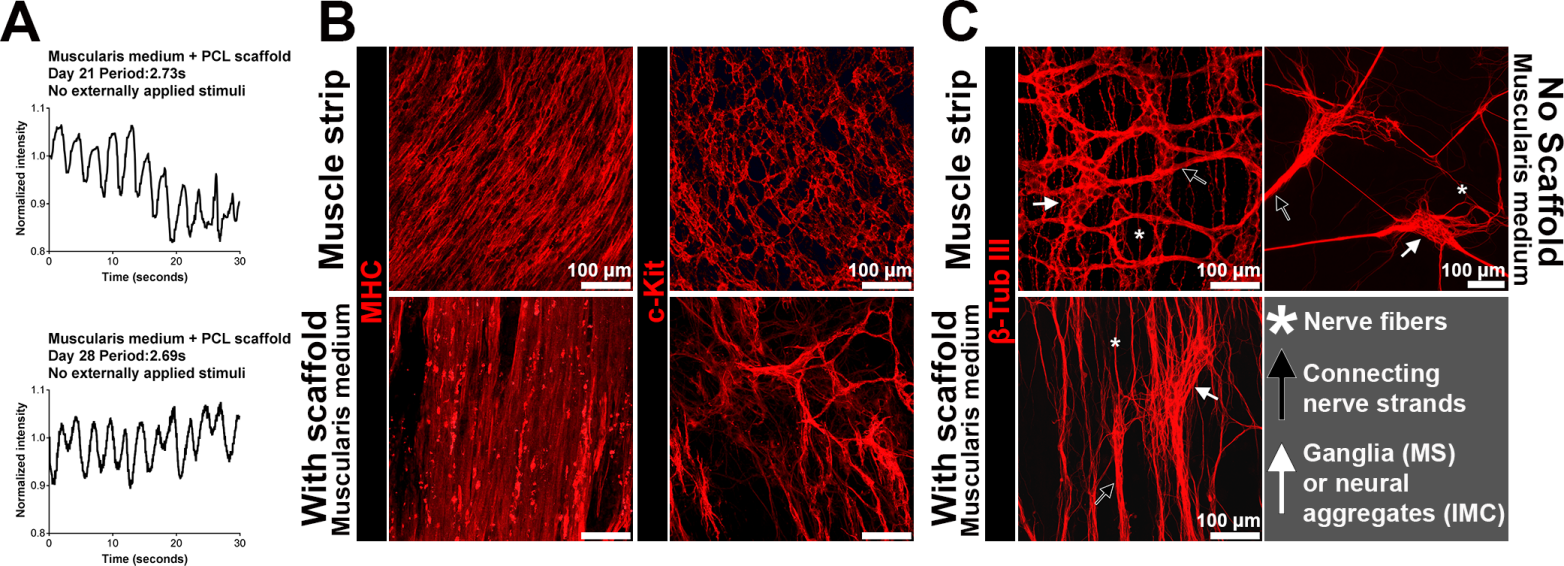
Spontaneous periodic contractions of IMC sheets on aligned electrospun PCL scaffolds in the muscularis medium. (**A**) Typical recordings of spontaneous periodic contractions of IMC sheets on PCL scaffolds in the muscularis medium (n = 3 biologically independent samples, at room temperature, 22 to 25°C, **S17 Video**). (**B**) Top views of mature smooth muscle cells (MHC) and ICC networks (c-Kit) in muscle strips and in IMC cultured on PCL scaffold in the muscularis medium at day 28, showing microarchitecture of muscle and ICC (confocal images, for MHC staining on muscle strips, mainly the circular muscle layer). (**C**) Top views of neurons (β-tubulin III) in muscle strips, IMC cultured on PCL scaffold and on culture plastic in the muscularis medium at day 28, showing aligned microarchitecture of neurons (confocal images for muscle strips and IMC on scaffolds). Key elements of myenteric plexus are pointed out: ganglia in muscle strips (MS) or ganglia-like neural aggregates in cultured IMC (white arrows), thick neurite bundles (black arrows) and neural fibers (white asterisks). Scale bars, 100 μm.

The IMC culture in the muscularis medium can be combined with other technologies for applications in intestinal regeneration. To guide IMC for more organized structure, we incorporated an aligned electrospun poly-caprolactone (PCL) sheet into the culture system. In the muscularis medium, the PCL sheets seeded with IMC periodically moved due to spontaneous contractions of IMC (**Fig 6A**, **S17 Video**, n = 3 bio-independent samples). MHC^+^ smooth muscle cells and β-tubulin III^+^ neuronal plexus lined up along with the PCL fiber structure, while the ICC formed a rudimentary network (**Fig 6B and 6C**).

### Muscularis medium supported both epithelium and IMC with contractions

**Fig 7:**
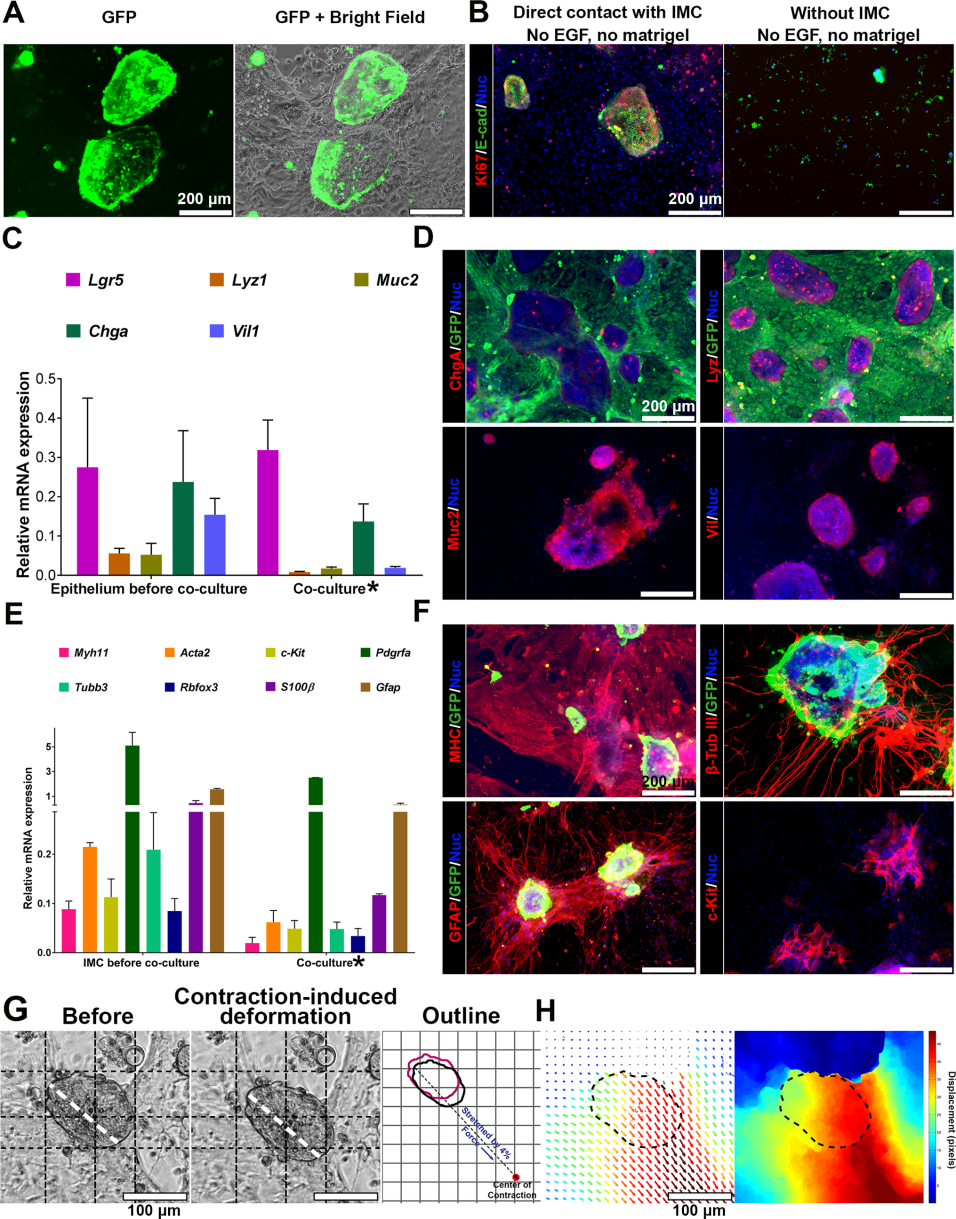
Intestinal epithelium and functional IMC both survive in the muscularis medium. (**A**) GFP epithelium after 4-day co-culture with non-GFP IMC. (**B**) Proliferation (Ki67, red) of epithelium (E-cadherin, E-cad, green) when cultured alone or with IMC. (**C**) Relative mRNA expression of indicated epithelial markers of epithelium before co-culture and cells after 4-day co-culture*. (**D**) Immunofluorescence of ChgA, Lyz, Muc2, Vil and GFP for GFP IMC and non-GFP epithelium after 4-day co-culture. (**E**) Relative mRNA expression of indicated IMC markers of IMC before co-culture and cells after 4-day co-culture*. (**F**) Immunofluorescence of MHC, c-Kit, β-tubulin III, GFAP and GFP for GFP epithelium and non-GFP IMC after 4-day co-culture. (**G**) One representative epithelial cell cluster in co-culture (**S18 Video**) before (left) and after (middle) being stretched and the outlines (right image; before: magenta; after: black; grid, 50 μm; dashed line indicates the direction of IMC contraction; the number 4% was a reflection of the length change of the cluster before and after stretching along the direction of IMC contraction). (**H**) Optical-flow analysis of the same epithelial cell cluster in (**G**). The direction and length of arrows represent the direction and magnitude of the displacement at each location. The heat map is to visualize the magnitude of displacement of each pixel as the epithelial cell cluster being stretched. Dashed line outlines the area of the epithelial cell cluster before being stretched. RT-PCR in (**C**, **E**). Control: crypts (**C**), muscle strips (**E**); housekeeping gene: *Gapdh*. Error bars, S.D. (n = 3 biologically independent samples). Scale bars, 200 μm (**A**-**B**, **D**, **F**); 100 μm (**G**-**H**). DAPI (blue) for nuclei in (**B**, **D**, **F**). *The mRNA level in co-culture was normalized to *Gapdh* expressed by **all** cells (both epithelium and IMC) in co-culture. However, most of the epithelial or IMC markers (except *c-Kit*, *Chga* and *Pdgfra*) were mainly expressed by epithelial cells or IMC respectively. Therefore the mRNA level showed here for co-culture is artificially lower.

Additionally, when incorporated with the culture technology[39] of intestinal epithelium, the muscularis medium can also support the co-culture of both epithelium and functional IMC (**Fig 7A**, n = 6 bio-independent samples). In conventional EC medium, the growth of epithelium required exogenous EGF[39]. Without exogenous EGF and matrigel, almost no epithelial cells from the crypts could proliferate (**Fig 7B**, n = 3 bio-independent samples). Interestingly, when directly co-cultured with IMC, even without exogenous EGF and matrigel, epithelium in the muscluaris medium did proliferate (Ki67^+^ cells, **Fig 7B**, n = 3 bio-independent samples), suggesting IMC could mediate the proliferation pattern of epithelium. In direct co-culture, the epithelium contained a variety of cell types including enterocytes (*Vil1*), goblet cells (*Muc2*), enteroendocrine cells (*ChgA*), Paneth cells (*Lyz1*) and the epithelial stem cells (*Lgr5*, **Fig 7C**, n = 3 bio-independent samples). Immunofluorescence (n = 3 bio-independent samples) showed the co-expression of chromogranin A (Chga), Mucin 2 (Muc 2), lysozyme (Lyz) and villin (Vil, **Fig 7D**). IMC in direct co-culture continued to contract and expressed various markers of normal muscularis (**Fig 7E**, n = 3 bio-independent samples). Immunofluorescence (n = 3 bio-independent samples) further confirmed the presence of mature smooth muscle cells and the network of ICC (**Fig 7F**). Neurons and glial cells in direct co-culture retained the histotypic organization of the enteric ganglia-like neural aggregates, with interconnecting strands and dense mesh of outgrowing processes (**Fig 7F**). In addition, serosal mesothelial cells also existed in co-culture (**S6 Fig**).

IMC contractions also persisted in direct co-culture (**S18 Video**, n = 6 bio-independent samples). Epithelial cells mechanically interacted with IMC. Driven by the stress gradient, epithelial cells in direct co-culture were periodically stretched by the contracting IMC (**Fig 7G**, **S18 Video**). The stress gradient was reflected by the non-uniform displacements within one epithelial cluster (**Fig 7H**). The degree of strain was affected by the size of the epithelial structures and their relative location to the contracting IMC.

### Contractions of human IMC & human epithelium-IMC co-culture

**Fig 8:**
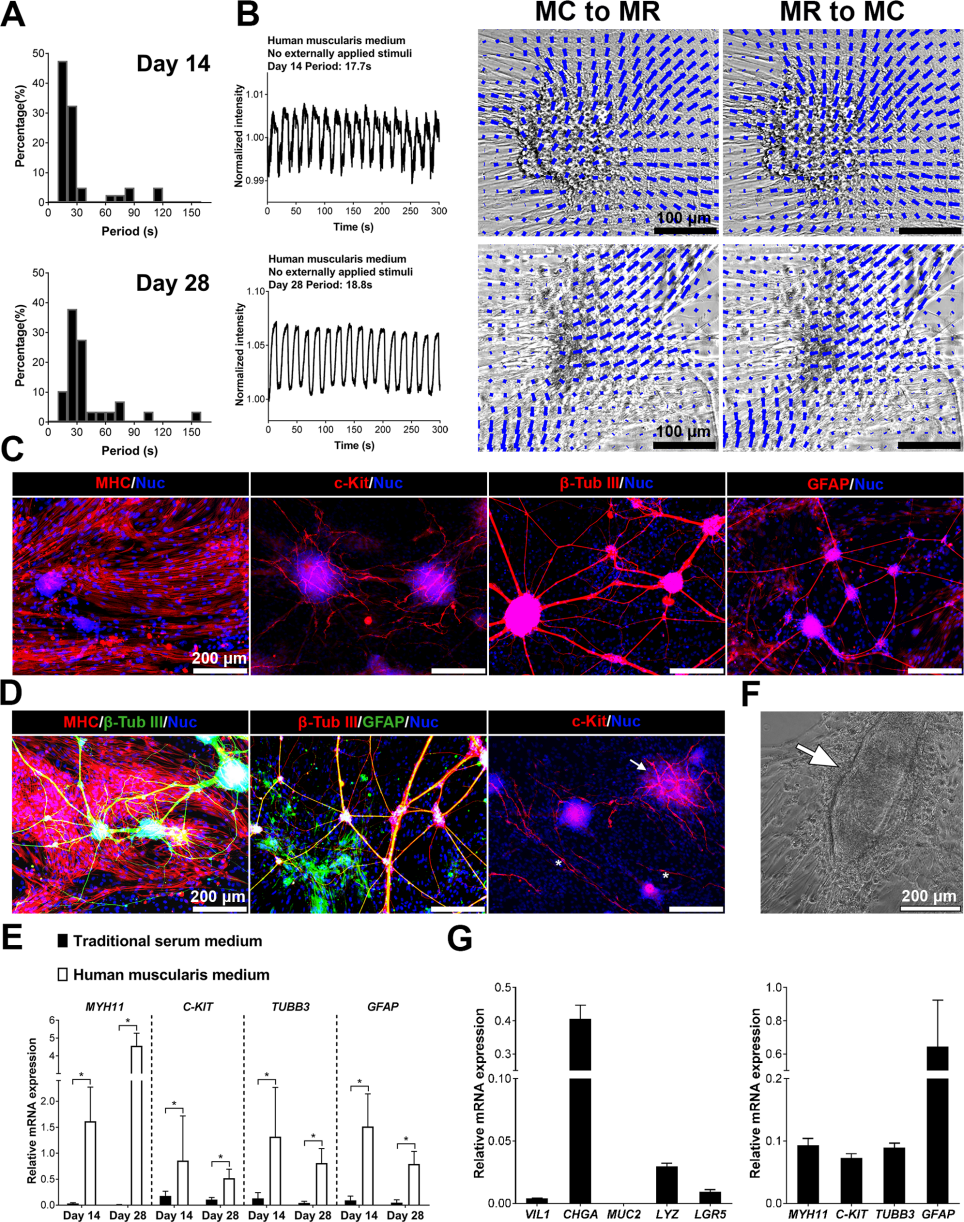
Contractility and cellular maturation of human fetal IMC and human IMC-epithelium co-culture. (**A**) Distributions of contraction periods of human fetal IMC in the human muscularis medium at day 14 (*40*, **3**; N = 40 cell clusters from n = 3 independent biological samples) and 28 (*29*, **3**). Spontaneous contractions (no stimulation, at room temperature, 22 to 25°C). (**B**) Recordings of spontaneous periodic contractions of one cell cluster in the human muscularis medium at day 14 and 28 (left). Images (right) of the same cluster from maximum contraction state to maximum relaxation state (MC to MR) and vice versa (MR to MC). See **S19 Video**. The direction and magnitude of the displacement at each location are indicated by the direction and length of each blue vector. Scale bars, 100 μm. (**C**) Immunofluorescence of MHC, c-Kit, β-tubulin III, and GFAP in the human muscularis medium at day 28. (**D**) Mature smooth muscle cells and neurons (left); neurons and glial cells co-localized to form neural networks (middle); multipolar ICC network (right, arrow) and dipolar ICC (right, asterisks). (**E**) Relative mRNA expression of indicated markers of human fetal IMC in the traditional serum and human muscularis media at day 14 and 28. Control: human fetal muscle strips; Housekeeping gene: *GAPDH*. Error bars, S.D. (n = 9 wells from 3 independent human samples). Two-tailed Student’s t-test, *p < 0.05. (**F**) IMC and human epithelium (arrow) in co-culture. (**G**) Relative mRNA expression of indicated epithelial or IMC markers of cells after 4-day co-culture. Control: Human crypts or human muscle strips, housekeeping gene: *GAPDH* (see note* in **Fig 7**). Error bars, S.D. (n = 3 wells). Scale bars in (**C**-**D**, **F**), 200 μm.

To realize the full potential of our new IMC system, we next investigated the capability of the muscularis medium for human cells. We noted that one component in the muscularis medium, N-acetyl-L-cysteine (Nac), can protect neurons against apoptosis[51] but induces apoptosis of smooth muscle cells[52]. Though Nac in the muscularis medium did not bring substantial damage of murine smooth muscle cells; for human smooth muscle cells, Nac considerably limited their survival and consequently attenuated human IMC contractility (**S7 Fig**). Upon removal of Nac, human fetal and postnatal IMC in this new medium (human muscularis medium) formed similar muscularis complexes with visible, spontaneous and periodic contractions (**Fig 8A and 8B**; **S8 Fig**, **S19 Video**, n = 3 (postnatal) or 6 (fetal) bio-independent samples). The periods of contractions for human fetal IMC clustered around 10–30 seconds at day 14 and 10–40 seconds at day 28 (**Fig 8A and 8B**, n = 3 bio-independent samples), which was similar to those of human fetal muscle strips (**S19**and S**20 Videos**, for muscle strips, n = 3 bio-independent samples). In addition, compared with the previous serum medium, the human muscularis medium also strongly supported the growth of mature smooth muscle cells, ICC, neurons and glial cells (**Fig 8C**–**8E**, n = 3 bio-independent samples). In this medium, mature smooth muscle cells distributed throughout the whole culture area; neurons and glia again co-localized to form structures reminiscent of the native myenteric plexus; networks formed by multipolar ICC were associated with the neural aggregates; while dipolar ICC resided along with the smooth muscle cells (**Fig 8C and 8D**). Directly co-cultured human epithelium and IMC also survived in human muscularis medium (**Fig 8F and 8G**, n = 3 wells). IMC exhibited rhythmic contractions with epithelium attached on top (**S21 Video**, n = 3 bio-independent samples).

## Discussion

Both human and murine IMC cultured in our serum-free media (muscularis medium and human muscularis medium) possess many features that are not achievable under conventional serum-containing conditions. Contractions of IMC in traditional serum cultures are transitory and irregular[5,6,46], in most cases, relying on external stimuli[5,46]. In contrast, contractions of murine and human fetal IMC in our media are 1) spontaneous (no stimulation), 2) periodic, 3) long-term, 4) with distinct physical movements and 5) with a frequency closely resembling that of native smooth muscle (murine at day 28; human fetal from day 14 to day 28; **Figs 1B, 1C**, **8A and 8B**; **S1**–**S3**, **S19**and S**20 Videos**). For human postnatal IMC, contractions in the human muscularis media are spontaneous, periodic but slower than those associated with fetal IMC. Researchers have shown that the frequency of contractions will increase when temperature is raised[11]. In this report, all the contraction frequency tests, both for IMC and muscle strips, were conducted at room temperature (22°C to 25°C). Cells and tissues may contract faster at 37°C.

For an extended period, all critical cell populations from intestinal muscularis, including mature smooth muscle cells, neural networks and ICC, not only survived but retained their histotypic morphology. The discovery of neurons, glia and ICC with different morphologies implies that the (human) muscularis media can preserve the microenvironment for regional specialization. Furthermore, the formation of the neural networks that share many common morphological features with the native myenteric plexus suggests that the muscularis media can also maintain the unique cell-cell associations within intestinal muscularis complexes. The inability of reverting intestinal smooth muscle cells into the mature phenotype *in vitro* has been often discussed in the literature[18,19,53]. Here we reverted non-contractile smooth muscle cells to the mature contractile phenotype by culturing them in the (human) muscularis media. Studies have suggested that neuron-smooth muscle cell interactions are essential for developments of both smooth muscle and ENS[5]. The acquisition of maturity for smooth muscle cells, ICC, neurons and glial cells in the (human) muscularis media may be a result of the close connectivity among these cells.

The serum-free muscularis media also offer a defined environment for mechanistic studies (**S2 Note**, **S22 Video**). In the muscularis medium, different components in combination displayed a potent synergistic effect on IMC contractility. While simpler formulations of serum-free media can be used, the efficiency of periodic contractions was reduced (**S1 Note**). In particular, we observed a marked decline of *c-Kit* expression when noggin, R-spondin1 and Y27632 were omitted (**S9 Fig**, n = 3 bio-independent samples), suggesting pathways controlled by these three components may modulate the growth and maturation of ICC.

The muscularis medium is ready to complement alternative techniques for applications in intestinal regeneration. Particularly, polymer scaffold with aligned structure is capable of providing sufficient topological cues for IMC culture. With aligned PCL scaffolds, IMC in the muscularis medium displayed enhanced architecture with more organized contractile movements. Previously, IMC have also been cultured on the similar PCL scaffold in the traditional serum medium, in which IMC formed similar aligned morphology but demonstrated no distinct movement[54]. These findings highlight the indispensable role of the muscularis medium for the functional regeneration of IMC sheets. The intestine has both circular and longitudinal muscle layers, future design of the scaffolds should have the similar multi-layer structure to better mimic the native tissue. In addition, smooth muscle cells, neurons and ICC may have different requirements regarding the substrate stiffness and composition. Therefore a mixture of different materials may be desirable in order to fulfill various biological and mechanical needs. Moreover, special factors for vasculature may also be integrated into the scaffold. With such optimizations of the scaffolds, the functional IMC sheets may eventually become the replacement tissue for treatment of related intestinal diseases.

Intestinal tissue engineering[55–57] and strategies of intestinal replacement require regeneration of both functional epithelium and muscularis under defined serum-free conditions. With the addition of intestinal epithelium, the new media here can support not only cells from muscularis, but also up to 11 different cell types from mucosa, muscularis and serosa (**Figs 7 and 8**, **S6 Fig**). Our culture system may serve as a platform for more complex and comprehensive studies of other cell types as well. In addition, peristalsis normally results in periodic waves of both muscularis and mucosa. The active mechanical factor is crucial to normal tissue physiology but always missing in traditional culture systems. Although the architecture of the co-culture requires further optimization, our co-culture system has recapitulated the cyclic mechanical strains by the natural contraction of IMC and re-established this coupled mechanical relation between epithelium and muscularis.

In summary, this is the first report of a platform that successfully maintains long-term spontaneous and periodic contractions of primary-cultured IMC in defined, serum-free conditions. The method can be broadly used and may ultimately assist in the full-thickness regeneration of functional intestine when in combination with other various technologies such as bio-scaffold and culture methods for intestinal epithelium. The new media can even be used together with the pluripotent stem cell-based culture method[4] to help identify the pathways that promote the intestinal fate. Contractile motion of smooth muscle is essential for intestinal barrier function[58]. IMC culture in the new media may also be of unique value when introduced into the gut-on-a-chip micro-devices for studies of microbiome-host interactions[58]. The serum-free cultures described here provide a more realistic model for utilization in therapeutic testing and future mechanistic studies of gut motility disorders. The findings from this new culture may also shed light on the regeneration of other organs in the gastrointestinal tract.

## Supporting information

S1 Fig Contractions of cell clusters were represented by the intensity change. (**A**) Averaged fluorescence intensity of regions within the white boxes increased when the cell clusters contracted and decreased when the cell clusters relaxed. (**B**) For non-GFP cells in phase contrast videos, the mean intensity decreased (darker) as the cell cluster turned into contraction state (red boxes). Scale bars, 200 μm. (**C**) The cell cluster contracted to reveal the background at the edge (white boxes). Scale bars in (**A**, **C**), 100 μm.
(PDF)

S2 Fig Drug vehicles (distilled water and DMSO) had little effect on the contraction frequency of IMC in the muscularis medium. (**A**) Representative recordings of the immediate effect of distilled water on IMC in the muscularis medium at d28 (n = 3 biologically independent samples). (**B**) Representative recordings of the effect of distilled water on IMC in the muscularis medium at d28 after a 3-min incubation at 37°C (n = 3 biologically independent samples). (**C**) Representative recordings of the effect of DMSO on IMC in the muscularis medium at d28 after a 15-min incubation at 37°C (n = 3 biologically independent samples). Seven different drugs were used in this study, including carbachol, SNP, DMPP, hexamethonium, L-NAME, TTX and niflumic acid. All of the drugs were dissolved in distilled water, except niflumic acid in DMSO. The water solution of carbachol, DMPP and hexamethonium had an immediate effect on IMC, while SNP, L-NAME and TTX required a 3 to 5-min incubation at 37°C before showing a steady effect. We then tested the immediate effect of water (**A**) and its later effect after a 3-min incubation at 37°C (**B**). For niflumic acid dissolved in DMSO, IMC was incubated with the drug solution for 15 mins at 37°C prior to video recording. Here we tested the DMSO effect after the 15-min incubation at 37°C (**C**).
(PDF)

S3 Fig IMC in the EC medium displayed neurites-like structure. Representative GFP fluorescence image of murine IMC in EC medium at day 7. The arrow indicates the neurites-like fibers in culture. Scale bar, 200 μm.
(PDF)

S4 Fig Contractions of IMC at early time points in the muscularis medium. Distributions of contraction periods of IMC in the muscularis medium at day 7 (*59*, **6**; N = *59* cell clusters from n = **6** biologically independent samples) and 14 (*174*, **7**).
(PDF)

S5 Fig Effects of TTX on fresh muscle strips and IMC in the muscularis medium. (**A**) Representative recordings of the effect of TTX at 400 μM on IMC in the muscularis medium at d28, matching **S14 Video**. (**B**) Representative recordings of the effects of TTX at 10 μM, 400 μM and 1 mM on fresh muscle strips, matching **S15 Video**.
(PDF)

S6 Fig Serosal mesothelial cells also existed in epithelium-muscularis co-culture. Immunofluorescence of cytokeratin after 4-day co-culture of epithelium and IMC. Scale bar, 200 μm.
(PDF)

S7 Fig The presence of Nac substantially limited the survival of mature human smooth muscle cells. MHC (smooth muscle cells) and β-Tub III (neurons) staining of human fetal IMC in muscularis medium (with Nac) and human muscularis medium (without Nac) after 21-day culture. DAPI (blue) stained the nuclei. Scale bars, 500 μm.
(PDF)

S8 Fig Periodic contractions of human postnatal IMC in the human muscularis medium. (**A**) Recordings of periodic contractions of one human infant IMC cluster in the human muscularis medium at day 22 (**S19 Video**). (**B**) Recordings of periodic contractions of one human postnatal IMC cluster in the human muscularis medium at day 28 (left) and its phase contrast images at contraction and relaxation states (right), corresponding to **S19 Video**. Black arrows indicate the direction of movement. Scale bars, 50 μm. (**C**) Morphological difference between murine and human infant IMC at day 28 in the muscularis and human muscularis media, respectively. Similar to the contractions of murine IMC, contractions of human infant IMC were also initiated at the location of the cell clusters. In general, the human intestinal muscularis complexes were denser and smaller than the murine intestinal muscularis complexes. Scale bars, 200 μm.
(PDF)

S9 Fig The expression of c-Kit decreased when noggin, R-spondin1 and Y27632 (NRY) were removed from the muscularis medium. (**A**) Immunofluorescence of c-Kit at day 7 and 28 (n = 3 biologically independent samples). Scale bars, 200 μm. (**B**) Relative mRNA expression of *c-Kit* in the serum medium, muscularis medium and the medium without NRY at day 2 (pre-incubation in the serum medium), 7, 14, 28 and 56, measured by real-time RT-PCR. Muscle strips served as control, *Gapdh* as the housekeeping gene. Error bars, S.D. (n = 3 biologically independent samples). Experimental groups were compared by ANOVA and Tukey’s post hoc method. *p < 0.05.
(PDF)

S1 Table Antibodies, primers and probes used in the study. (PDF)

S2 Table Components in the EC medium and their possible functions in IMC culture. (PDF)

S3 Table Selected results of medium component assessment for IMC culture. (PDF)

S1 Note The development of the muscularis medium for IMC culture. (PDF)

S2 Note Rendering the culture condition totally serum-free. (PDF)

S1 Video Contractions of murine muscle strips (real time). Spontaneous periodic contractions of the non-GFP muscle strip (from a 5-day-old mouse) after 6-hour incubation in DMEM with ABAM at 37°C, corresponding to **Fig 1C**. Real time. Arrow indicates the spot tested to show the recording of intensity change in **Fig 1C**. N = 62 spots from n = 21 animals, and here only one representative sample is shown. Magnification, 40x. Contractile assessments were conducted at room temperature (22 to 25°C).
(MP4)

S2 Video Representative murine IMC contractions (real time). Two samples of spontaneously and periodically contracting murine IMC in the muscularis medium. They are biologically independent. Sample 1 is GFP IMC in the muscularis medium at day 19 (00:00 to 00:30, 30 seconds); Sample 2 is non-GFP IMC in the muscularis medium at day 28 (00:30 to 01:33, ~1 minute). Both real time. n = 80 biologically independent samples, and here only two representative ones are shown. Magnification, 40x. Contractile assessments were conducted at room temperature (22 to 25°C).
(MP4)

S3 Video IMC contractions d7-d56 (real time). Spontaneous and periodic contractions of GFP murine IMC in the muscularis medium at day 7, 14, 21, 28, 35, 42, 49 and 56, corresponding to **Fig 1B and 1C** and **S4 Fig**. Each about 30 seconds. All real time. All videos at different time points were taken from the same sample. n = 4 biologically independent samples, and here only one representative sample is shown. Magnification, 40x. Contractile assessments were conducted at room temperature (22 to 25°C).
(MP4)

S4 Video Contractions of passaged IMC (real time). Contractions of murine IMC initially cultured in the serum medium for 4 days then passaged and cultured for 14 days. Passaged cells were cultured in the muscularis medium. Real time. n = 3 biologically independent samples, and here only one representative sample is shown. Magnification, 40x. Contractile assessments were conducted at room temperature (22 to 25°C).
(MP4)

S5 Video Contractions of filtered IMC (real time). Spontaneous and periodic contractions of filtered murine IMC in the muscularis medium for 21 days. Real time. n = 3 biologically independent samples, and here only one representative sample is shown. Magnification, 40x. Contractile assessments were conducted at room temperature (22 to 25°C).
(MP4)

S6 Video No contractions in traditional serum medium (real time). Murine GFP IMC in the serum medium at day 7, 14, 21, 28, 35, 42, 49 and 56. No contractions were generated. Each about 30 seconds. All real time. All videos at different time points were taken from the same sample and this sample was from the same animals as the sample in **S3 Video**. n = 3 biologically independent samples, and here only one representative sample is shown. Magnification, 40x. Contractile assessments were conducted at room temperature (22 to 25°C).
(MP4)

S7 Video Carbachol effect, short and full versions (real time). Effects of carbachol on non-GFP murine IMC in the muscularis medium at day 28 (first a short version for a quick view of the effect, 00:00–00:47, then the full version, 00:47–04:05). **Short version:** First half (magnification, 40x): before adding carbachol (00:04–00:13), adding carbachol (00:13–00:16) and after adding 50 μM carbachol (00:16–00:24); second half (magnification, 100x): before adding carbachol (00:26–00:36), adding carbachol (00:36–00:40) and after adding 10 μM carbachol (00:40–00:47). **Full version:** First half (magnification, 40x): before adding carbachol (00:50–01:29), adding carbachol (01:29–01:32) and after adding 50 μM carbachol (01:32–02:33); second half (magnification, 100x): before adding carbachol (02:35–03:07), adding carbachol (03:07–03:10) and after adding 10 μM carbachol (03:10–04:05). Real time. n = 3 biologically independent samples for each concentration, and here only one representative sample for each concentration is shown. CCh, carbachol. Contractile assessments were conducted at room temperature (22 to 25°C).
(MP4)

S8 Video Carbachol effect, muscle strips (real time). Effects of carbachol on murine muscle strips (after 6-hour incubation in DMEM with ABAM at 37°C). **First half:** before adding carbachol (00:02–00:39), adding carbachol (00:39–00:50) and after adding 50 μM carbachol (00:50–01:45); **second half:** before adding carbachol (01:47–02:15), adding carbachol (02:15–02:25) and after adding 10 μM carbachol (02:25–03:19). Real time. n = 3 animals for each concentration, and here only one representative sample for each concentration is shown. Magnification, 40x. Contractile assessments were conducted at room temperature (22 to 25°C).
(MP4)

S9 Video SNP effect (real time). Effects of 100 μM SNP on non-GFP murine IMC in the muscularis medium at day 28. The video captures the same spot before (00:00–01:00) and after (01:00–02:00) adding 100 μM SNP in culture. Arrow indicates the cell cluster tested to show the recording of intensity change in **Fig 4A**. Both real time. n = 3 biologically independent samples, and here only one representative sample is shown. Magnification, 40x. Contractile assessments were conducted at room temperature (22 to 25°C).
(MP4)

S10 Video Niflumic acid effect (real time). Effects of 300 μM niflumic acid on non-GFP murine IMC in the muscularis medium at day 35. The video captures the same spot before (00:00–00:44) and after (00:44–01:29) adding 300 μM niflumic acid in culture. Arrow indicates the cell cluster tested to show the recording of intensity change in **Fig 4C**. Both real time. n = 3 biologically independent samples, and here only one representative sample is shown. Magnification, 40x. Contractile assessments were conducted at room temperature (22 to 25°C).
(MP4)

S11 Video Niflumic acid effect, muscle strips (real time). Effects of 300 μM niflumic acid on non-GFP murine muscle strips (after 6-hour incubation in DMEM with ABAM at 37°C). The video captures the same muscle strip before (00:00–00:45) and after (00:45–01:30) adding 300 μM niflumic acid. Both real time. n = 6 animals, and here only one representative sample is shown. Magnification, 40x. Contractile assessments were conducted at room temperature (22 to 25°C).
(MP4)

S12 Video Calcium oscillations (real time). Spontaneous periodic Ca^2+^ oscillations of murine IMC in the muscularis medium for 28 days. Arrow indicates the cell cluster tested to show the recording of intensity change in **Fig 4D**. Real time. n = 3 biologically independent samples, and here only one representative sample is shown. Magnification, 100x. Contractile assessments were conducted at room temperature (22 to 25°C).
(MP4)

S13 Video Calcium propagation (real time). Ca^2+^ flux propagation in murine IMC cultured in the muscularis medium for 28 days, corresponding to **Fig 4E**. Real time. n = 3 biologically independent samples, and here only one representative sample is shown. Magnification, 100x. Contractile assessments were conducted at room temperature (22 to 25°C).
(MP4)

S14 Video TTX effect (speed 2x). Effects of TTX on non-GFP murine IMC in the muscularis medium at day 28. **First half:** before (00:00–00:23) and after (00:23–00:45) adding 10 μM TTX in culture. **Second half:** before (00:46–01:09) and after (01:09–01:31) adding 400 μM TTX in culture. Arrows indicate the cell clusters tested to show the recording of intensity change in **Fig 5A** and **S5 Fig**. n = 6 biologically independent samples for each concentration, and here only one representative sample for each concentration is shown. Magnification, 40x. This video is at 2x speed. Contractile assessments were conducted at room temperature (22 to 25°C).
(MP4)

S15 Video TTX effect, muscle strips (speed 2x). Effects of TTX on non-GFP murine muscle strips (after 6-hour incubation in DMEM with ABAM at 37°C). **First 1/3:** before (00:01–00:23) and after (00:23–00:45) adding 10 μM TTX. **1/3 to 2/3:** before (00:46–01:09) and after (01:09–01:31) adding 400 μM TTX. **2/3 to end:** before (01:33–01:55) and after (01:55–02:17) adding 1 mM TTX. Arrows indicate spots tested to show the recording of intensity change in **S5 Fig**. n = 6 animals for each concentration, and here only one representative sample for each concentration is shown. Magnification, 40x. This video is at 2x speed. Contractile assessments were conducted at room temperature (22 to 25°C).
(MP4)

S16 Video Effects of DMPP, DMPP with Hexamethonium and DMPP with L-NAME (speed 2x). Effects of 10 μM DMPP, 10 μM DMPP with 300 μM hexamethonium or 10 μM DMPP with 100 μM L-NAME on non-GFP murine IMC in the muscularis medium at day 28. **DMPP, 00:00–00:34:** Before (00:00–00:17) and after (00:17–00:35) adding 10 μM DMPP in culture. Arrow indicates the cell cluster tested to show the recording of intensity change in **Fig 5B**. n = 6 biologically independent samples, and here only one representative sample is shown. **DMPP with Hexamethonium, 00:35–01:09:** Before (00:35–00:52) and after (00:52–01:09) adding 10 μM DMPP simultaneously with 300 μM hexamethonium in culture. Arrow indicates the cell cluster tested to show the recording of intensity change in **Fig 5C**. n = 3 biologically independent samples, and here only one representative sample is shown. **DMPP with L-NAME, 01:09–01:44:** Before (01:09–01:27) and after (01:27–01:44) adding 10 μM DMPP with a 3-min pre-treatment of 100 μM L-NAME in culture. Arrow indicates the cell cluster tested to show the recording of intensity change in **Fig 5D**. n = 3 biologically independent samples, and here only one representative sample is shown. Magnification, 40x. This video is at 2x speed. Contractile assessments were conducted at room temperature (22 to 25°C).
(MP4)

S17 Video IMC on scaffold (real time). Spontaneously and periodically contracting murine IMC sheets on PCL scaffolds in the muscularis medium at day 21(00:00–00:30) and day 28 (00:30–01:00), matching the recording of intensity change in **Fig 6A**. Both real time. n = 3 biologically independent samples, and here only one representative sample is shown. Magnification, 40x. Contractile assessments were conducted at room temperature (22 to 25°C).
(MP4)

S18 Video Epithelium moving with IMC in co-culture (real time). The movement of murine intestinal epithelium and IMC in the muscularis medium after co-cultured for 4 days. The arrow and asterisk indicate two epithelial clusters. The arrow points to the epithelial cell cluster tested to show the deformation in **Fig 7G and 7H**. Real time. n = 6 biologically independent samples, and here only one representative sample is shown. Magnification, 100x. Contractile assessments were conducted at room temperature (22 to 25°C).
(MP4)

S19 Video Contractions of human IMC (speed 20x). Spontaneous (no stimulation) and periodic contractions of human IMC in the human muscularis medium. **First half:** 16-week-old human fetal IMC in the human muscularis medium. (Sample 1 at day 14: 00:03–00:17; Sample 1 at day 28: 00:17–00:32; Sample 2 at day 14: 00:32–00:47). Sample 1 is the same muscularis complex shown in **Fig 8B**. n = 6 biologically independent samples. **Second half:** human IMC from a 12-year-old patient in the human muscularis medium at day 14 (00:50–01:04); human infant IMC in the human muscularis medium at day 22 (01:05–01:18) and day 28 (01:19–01:34), matching **S8A** and **S8B Fig**. Magnification, 200x. This video is at 20x speed. Contractile assessments were conducted at room temperature (22 to 25°C).
(MP4)

S20 Video Human muscle strips (speed 20x). Spontaneous and periodic contractions of the human fetal muscle strip (16-week-old) after 2-day incubation in DMEM with ABAM at 37°C. n = 3 biologically independent samples, and here only one representative spot is shown. Magnification, 40x. This video is at 20x speed. Contractile assessments were conducted at room temperature (22 to 25°C).
(MP4)

S21 Video Human epithelium moving with IMC (speed 20x). The movement of human intestinal epithelium and IMC in the human muscularis medium after co-cultured for 4 days. The arrow points to one epithelial cell cluster sitting on top of the contracting IMC. n = 3 biologically independent samples. Magnification, 100x. This video is at 20x speed. Contractile assessments were conducted at room temperature (22 to 25°C).
(MP4)

S22 Video BSA (real time). Spontaneous (no stimulation) and periodic contractions of non-GFP murine IMC in the muscularis medium at day 28, initial pre-incubation without serum, corresponding to **S2 Note**. Real time. n = 3 biologically independent samples, and here only one representative sample is shown. Magnification, 100x. Contractile assessments were conducted at room temperature (22 to 25°C).
(MP4)

S1 Code Contraction frequency test for GFP cells. (PDF)

S2 Code Contraction frequency test for GFP cells (strong background noise). (PDF)

S3 Code Contraction frequency test for non-GFP cells. (PDF)
