# Supplementary material for: Bioengineered intestinal muscularis complexes with long-term spontaneous and periodic contractions
Source: PLoS One. 2018 May 2;13(5):e0195315. doi: 10.1371/journal.pone.0195315 (PMC5931477; doi:10.1371/journal.pone.0195315)
Supplement: S1 Fig — (A) Averaged fluorescence intensity of regions within the white boxes increased when the cell clusters contracted and decreased when the cell clusters relaxed. (B) For non-GFP cells in phase contrast videos, the mean intensity decreased (darker) as the cell cluster turned into contraction state (red boxes). Scale bars, 200 μm. (C) The cell cluster contracted to reveal the background at the edge (white boxes). Scale bars in (A, C), 100 μm. (PDF) [file pone.0195315.s001.pdf]

## Supplementary figure S1

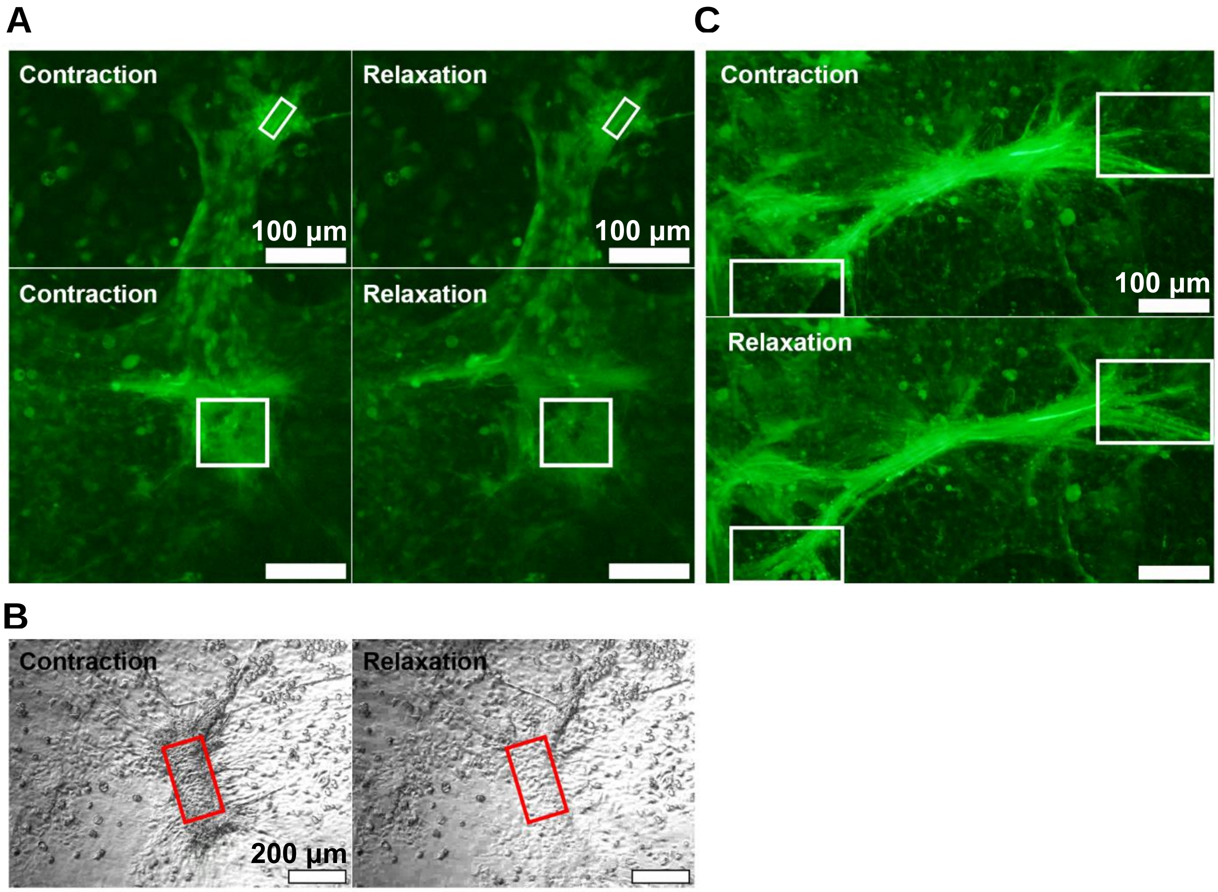


**S1 Fig. Contractions of cell clusters were represented by the intensity change.** (**A**) Averaged fluorescence intensity of regions within the white boxes increased when the cell clusters contracted and decreased when the cell clusters relaxed. (**B**) For non-GFP cells in phase contrast videos, the mean intensity decreased (darker) as the cell cluster turned into contraction state (red boxes). Scale bars, 200 µm. (**C**) The cell cluster contracted to reveal the background at the edge (white boxes). Scale bars in (**A**, **C**), 100 µm.
