# Supplementary material for: Bioengineered intestinal muscularis complexes with long-term spontaneous and periodic contractions
Source: PLoS One. 2018 May 2;13(5):e0195315. doi: 10.1371/journal.pone.0195315 (PMC5931477; doi:10.1371/journal.pone.0195315)
Supplement: S2 Fig — (A) Representative recordings of the immediate effect of distilled water on IMC in the muscularis medium at d28 (n = 3 biologically independent samples). (B) Representative recordings of the effect of distilled water on IMC in the muscularis medium at d28 after a 3-min incubation at 37°C (n = 3 biologically independent samples). (C) Representative recordings of the effect of DMSO on IMC in the muscularis medium at d28 after a 15-min incubation at 37°C (n = 3 biologically independent samples). Seven different drugs were used in this study, including carbachol, SNP, DMPP, hexamethonium, L-NAME, TTX and niflumic acid. All of the drugs were dissolved in distilled water, except niflumic acid in DMSO. The water solution of carbachol, DMPP and hexamethonium had an immediate effect on IMC, while SNP, L-NAME and TTX required a 3 to 5-min incubation at 37°C before showing a steady effect. We then tested the immediate effect of water (A) and its later effect after a 3-min incubation at 37°C (B). For niflumic acid dissolved in DMSO, IMC was incubated with the drug solution for 15 mins at 37°C prior to video recording. Here we tested the DMSO effect after the 15-min incubation at 37°C (C). (PDF) [file pone.0195315.s002.pdf]

Supplementary figure S2

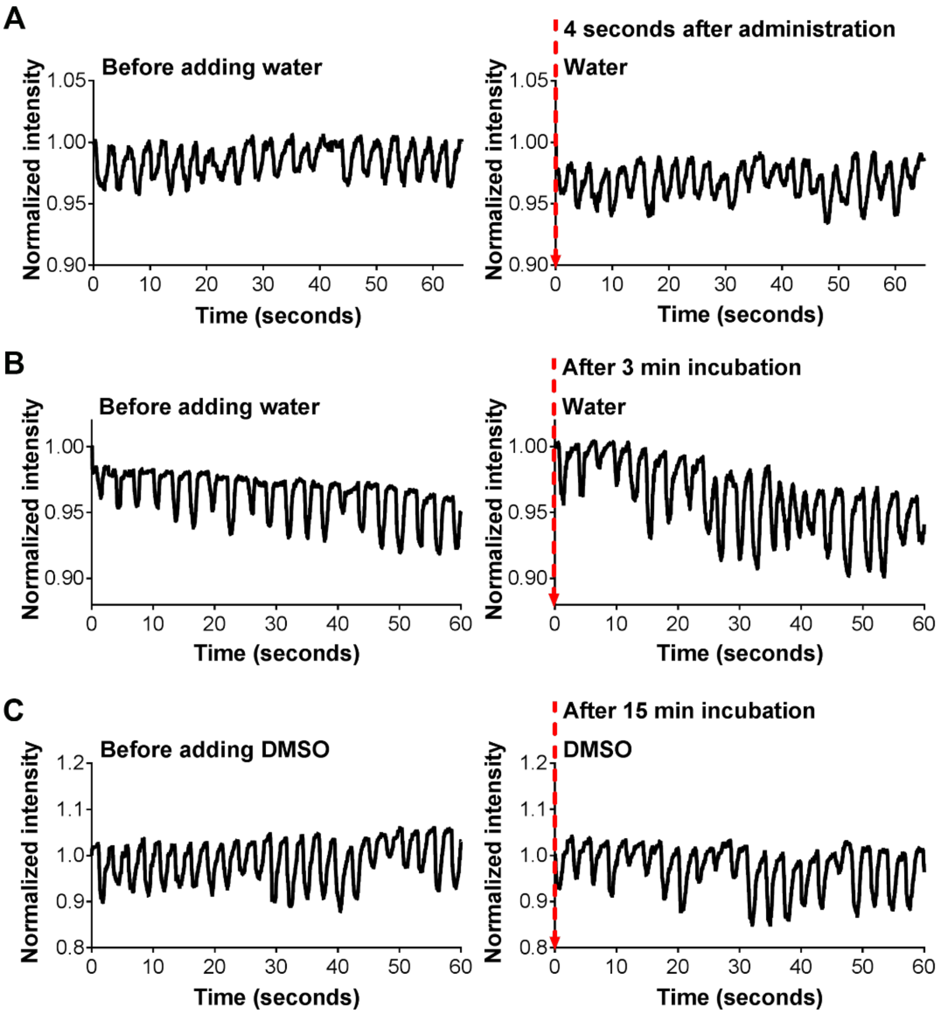

S2 Fig. Drug vehicles (distilled water and DMSO) had little effect on the contraction frequency of IMC in the muscularis medium. (**A**) Representative recordings of the immediate effect of distilled water on IMC in the muscularis medium at d28 (n = 3 biologically independent samples). (**B**) Representative recordings of the effect of distilled water on IMC in the muscularis medium at d28 after a 3-min incubation at 37°C (n = 3 biologically independent samples). (**C**) Representative recordings of the effect of DMSO on IMC in the muscularis medium at d28 after a 15-min incubation at 37°C (n = 3 biologically independent samples). Seven different drugs were used in this study, including carbachol, SNP, DMPP, hexamethonium, L-NAME, TTX and niflumic acid. All of the drugs were dissolved in distilled water, except niflumic acid in DMSO. The water solution of carbachol, DMPP and hexamethonium had an immediate effect on IMC, while SNP, L-NAME and TTX required a 3 to 5-min incubation at 37°C before showing a steady effect. We therefore tested the immediate effect of water (**A**) and its later effect after a 3-min incubation at 37°C (**B**). For niflumic acid dissolved in DMSO, IMC was incubated with the drug solution for 15 mins at 37°C prior to video recording. Here we tested the DMSO effect after the 15-min incubation at 37°C.
