# Supplementary material for: Bioengineered intestinal muscularis complexes with long-term spontaneous and periodic contractions
Source: PLoS One. 2018 May 2;13(5):e0195315. doi: 10.1371/journal.pone.0195315 (PMC5931477; doi:10.1371/journal.pone.0195315)
Supplement: S3 Fig — Representative GFP fluorescence image of murine IMC in EC medium at day 7. The arrow indicates the neurites-like fibers in culture. Scale bar, 200 μm. (PDF) [file pone.0195315.s003.pdf]

Supplementary figure S3

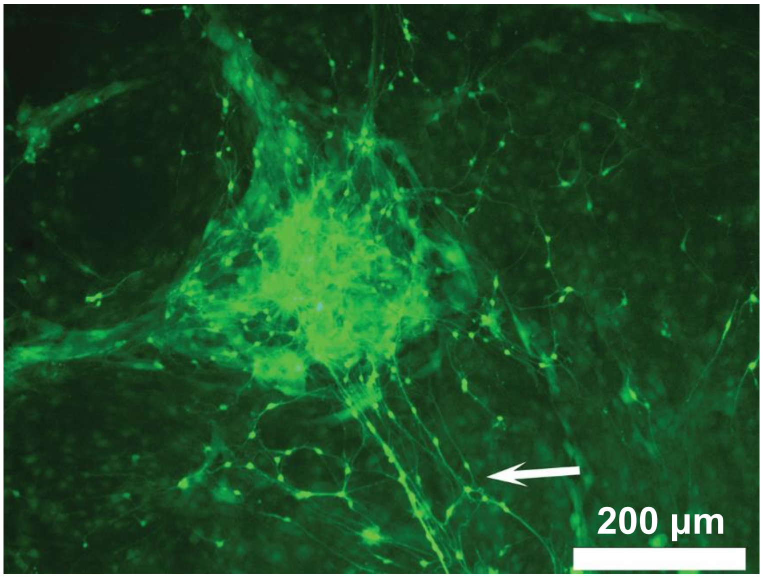

S3 Fig. IMC in the EC medium displayed neurites-like structure. Representative GFP fluorescence image of murine IMC in EC medium at day 7. The arrow indicates the neurites-like fibers in culture.
