# Supplementary material for: Bioengineered intestinal muscularis complexes with long-term spontaneous and periodic contractions
Source: PLoS One. 2018 May 2;13(5):e0195315. doi: 10.1371/journal.pone.0195315 (PMC5931477; doi:10.1371/journal.pone.0195315)
Supplement: S4 Fig — Distributions of contraction periods of IMC in the muscularis medium at day 7 (59, 6; N = 59 cell clusters from n = 6 biologically independent samples) and 14 (174, 7). (PDF) [file pone.0195315.s004.pdf]

Supplementary figure S4

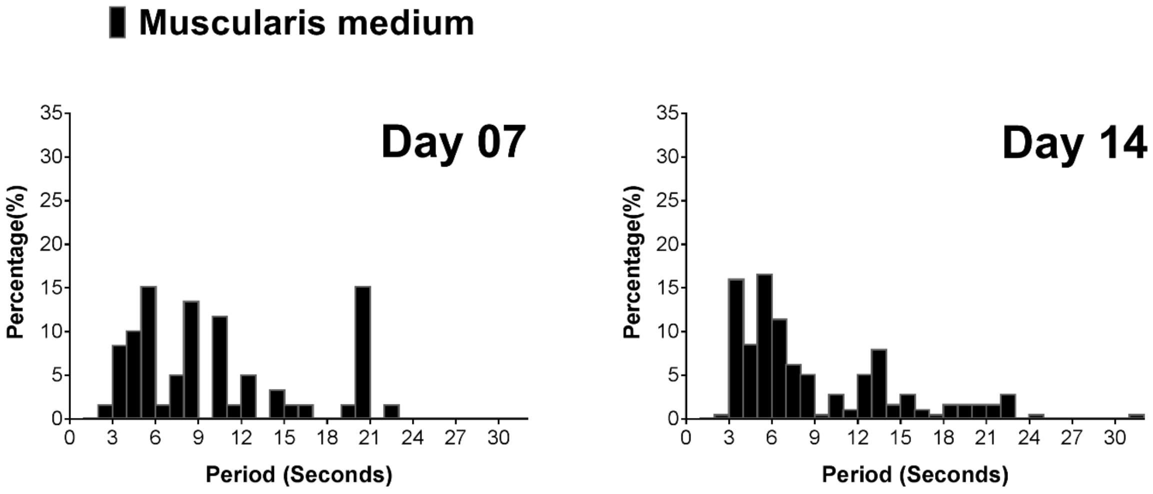

S4 Fig. Contractions of IMC at early time points in the muscularis medium. Distributions of contraction periods of IMC in the muscularis medium at day 7 (*59*, **6**; N = *59* cell clusters from n = **6** biologically independent samples) and 14 (*174*, **7**).
