# Supplementary material for: Bioengineered intestinal muscularis complexes with long-term spontaneous and periodic contractions
Source: PLoS One. 2018 May 2;13(5):e0195315. doi: 10.1371/journal.pone.0195315 (PMC5931477; doi:10.1371/journal.pone.0195315)
Supplement: S5 Fig — (A) Representative recordings of the effect of TTX at 400 μM on IMC in the muscularis medium at d28, matching S14 Video. (B) Representative recordings of the effects of TTX at 10 μM, 400 μM and 1 mM on fresh muscle strips, matching S15 Video. (PDF) [file pone.0195315.s005.pdf]

Supplementary figure S5

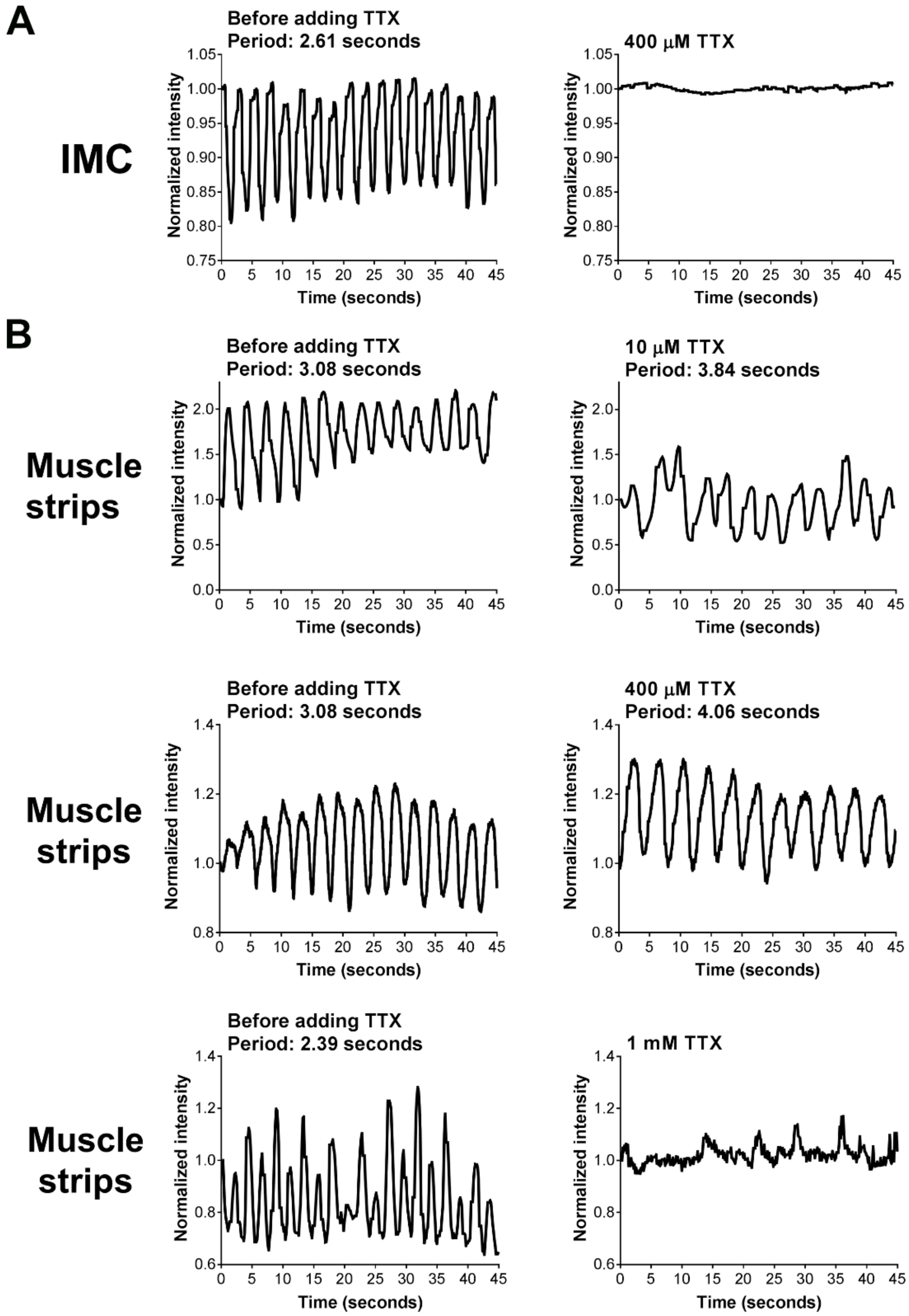

S5 Fig. Effects of TTX on fresh muscle strips and IMC in the muscularis medium. (**A**) Representative recordings of the effect of TTX at 400 μM on IMC in the muscularis medium at d28, matching **S14 Video**. (**B**) Representative recordings of the effects of TTX at 10 μM, 400 μM and 1 mM on fresh muscle strips, matching **S15 Video**.
