# Supplementary material for: Bioengineered intestinal muscularis complexes with long-term spontaneous and periodic contractions
Source: PLoS One. 2018 May 2;13(5):e0195315. doi: 10.1371/journal.pone.0195315 (PMC5931477; doi:10.1371/journal.pone.0195315)
Supplement: S6 Fig — Immunofluorescence of cytokeratin after 4-day co-culture of epithelium and IMC. Scale bar, 200 μm. (PDF) [file pone.0195315.s006.pdf]

Supplementary figure S6

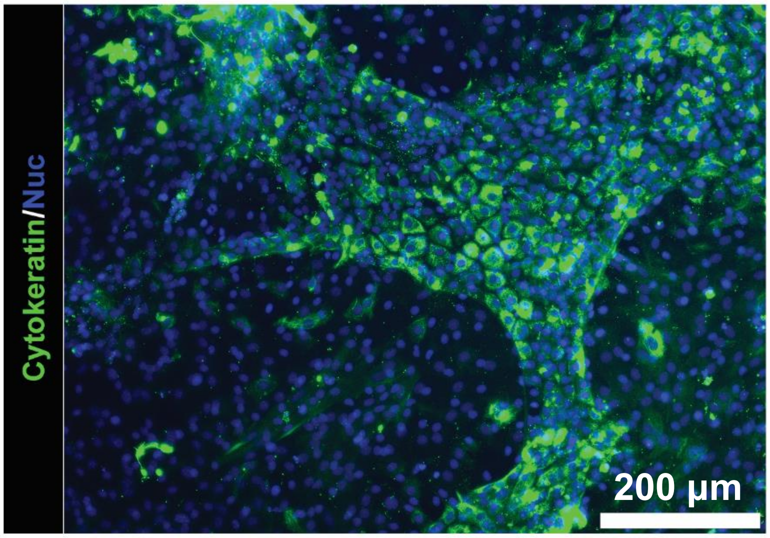

S6 Fig. Serosal mesothelial cells also existed in epithelium-muscularis co-culture. Immunofluorescence of cytokeratin after 4-day co-culture of epithelium and IMC.
