# Supplementary material for: Bioengineered intestinal muscularis complexes with long-term spontaneous and periodic contractions
Source: PLoS One. 2018 May 2;13(5):e0195315. doi: 10.1371/journal.pone.0195315 (PMC5931477; doi:10.1371/journal.pone.0195315)
Supplement: S7 Fig — MHC (smooth muscle cells) and β-Tub III (neurons) staining of human fetal IMC in muscularis medium (with Nac) and human muscularis medium (without Nac) after 21-day culture. DAPI (blue) stained the nuclei. Scale bars, 500 μm. (PDF) [file pone.0195315.s007.pdf]

Supplementary figure S7

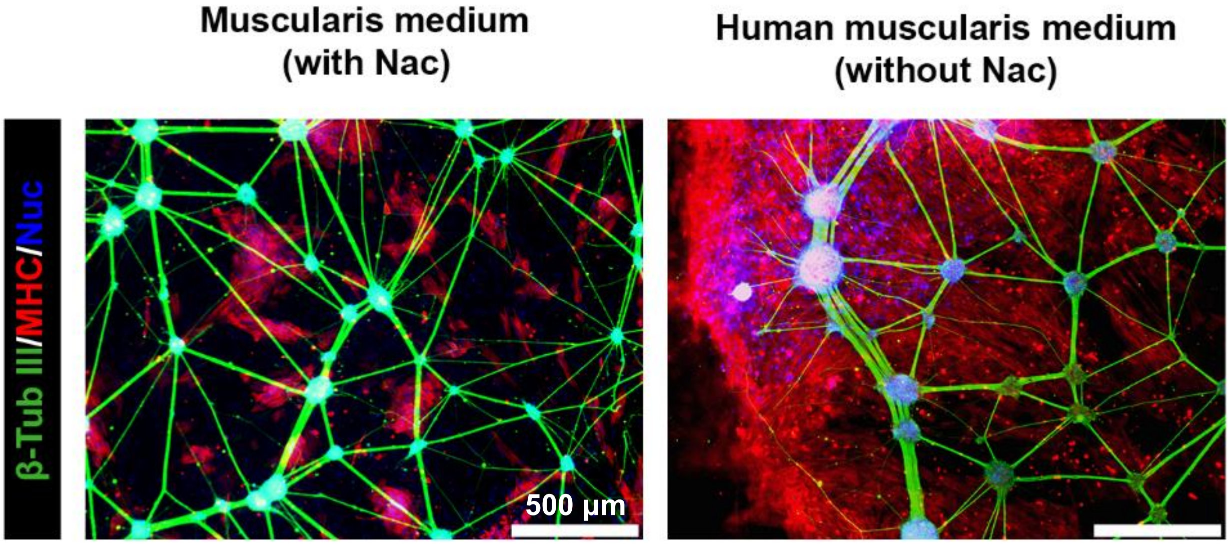

S7 Fig. The presence of Nac substantially limited the survival of mature human smooth muscle cells. MHC (smooth muscle cells) and β-Tub III (neurons) staining of human fetal IMC in muscularis medium (with Nac) and human muscularis medium (without Nac) after 21-day culture. DAPI (blue) stained the nuclei.
