# Supplementary material for: Bioengineered intestinal muscularis complexes with long-term spontaneous and periodic contractions
Source: PLoS One. 2018 May 2;13(5):e0195315. doi: 10.1371/journal.pone.0195315 (PMC5931477; doi:10.1371/journal.pone.0195315)
Supplement: S8 Fig — (A) Recordings of periodic contractions of one human infant IMC cluster in the human muscularis medium at day 22 (S19 Video). (B) Recordings of periodic contractions of one human postnatal IMC cluster in the human muscularis medium at day 28 (left) and its phase contrast images at contraction and relaxation states (right), corresponding to S19 Video. Black arrows indicate the direction of movement. Scale bars, 50 μm. (C) Morphological difference between murine and human infant IMC at day 28 in the muscularis and human muscularis media, respectively. Similar to the contractions of murine IMC, contractions of human infant IMC were also initiated at the location of the cell clusters. In general, the human intestinal muscularis complexes were denser and smaller than the murine intestinal muscularis complexes. Scale bars, 200 μm. (PDF) [file pone.0195315.s008.pdf]

Supplementary figure S8

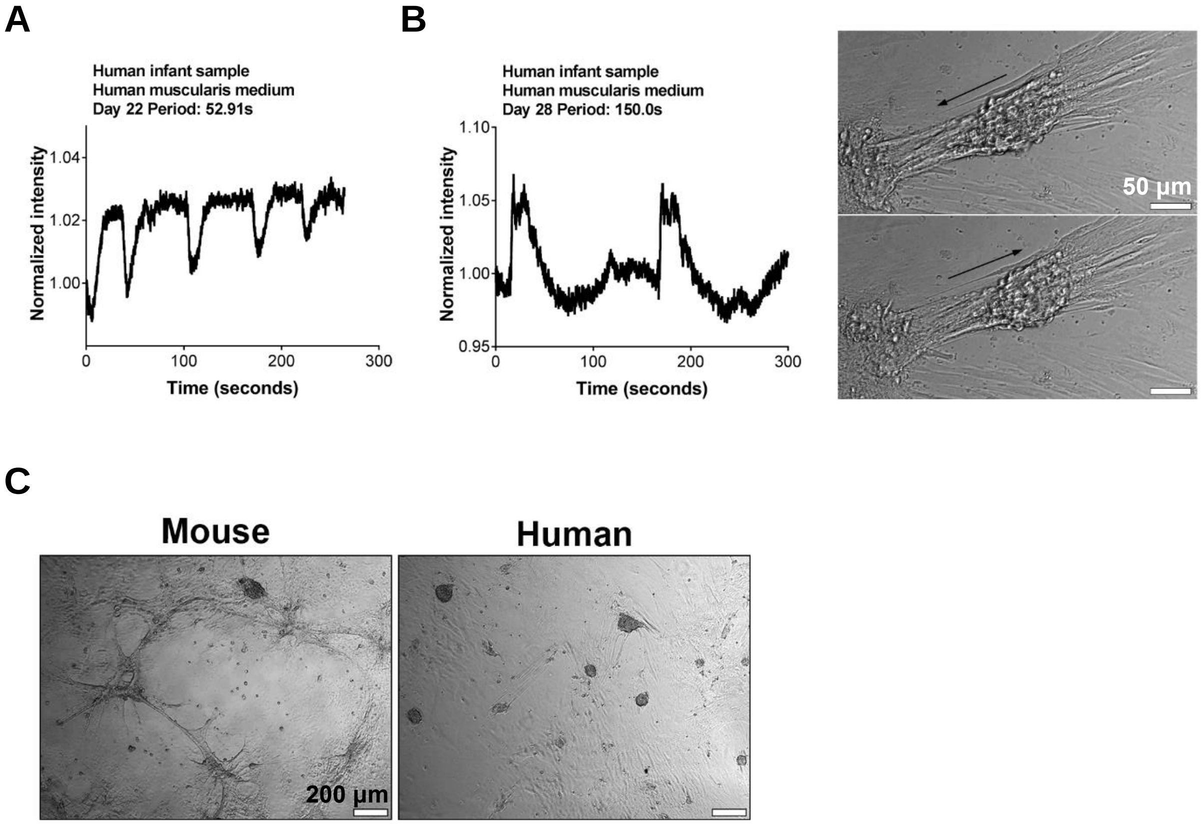

S8 Fig. Periodic contractions of human postnatal IMC in the human muscularis medium. (**A**) Recordings of periodic contractions of one human infant IMC cluster in the human muscularis medium at day 22 (**S19 Video**). (**B**) Recordings of periodic contractions of one human postnatal IMC cluster in the human muscularis medium at day 28 (left) and its phase contrast images at contraction and relaxation states (right), corresponding to **S19 Video**. Black arrows indicate the direction of movement. Scale bars, 50 µm. (**C**) Morphological difference between murine and human infant IMC at day 28 in the muscularis and human muscularis media, respectively. Similar to the contractions of murine IMC, contractions of human fetal IMC were mainly initiated at the location of the cell clusters. In general, human intestinal muscularis complexes were denser and smaller than the murine intestinal muscularis complexes. Scale bars, 200 µm.
