# Supplementary material for: Bioengineered intestinal muscularis complexes with long-term spontaneous and periodic contractions
Source: PLoS One. 2018 May 2;13(5):e0195315. doi: 10.1371/journal.pone.0195315 (PMC5931477; doi:10.1371/journal.pone.0195315)
Supplement: S9 Fig — (A) Immunofluorescence of c-Kit at day 7 and 28 (n = 3 biologically independent samples). Scale bars, 200 μm. (B) Relative mRNA expression of c-Kit in the serum medium, muscularis medium and the medium without NRY at day 2 (pre-incubation in the serum medium), 7, 14, 28 and 56, measured by real-time RT-PCR. Muscle strips served as control, Gapdh as the housekeeping gene. Error bars, S.D. (n = 3 biologically independent samples). Experimental groups were compared by ANOVA and Tukey’s post hoc method. *p < 0.05. (PDF) [file pone.0195315.s009.pdf]

Supplementary figure S9

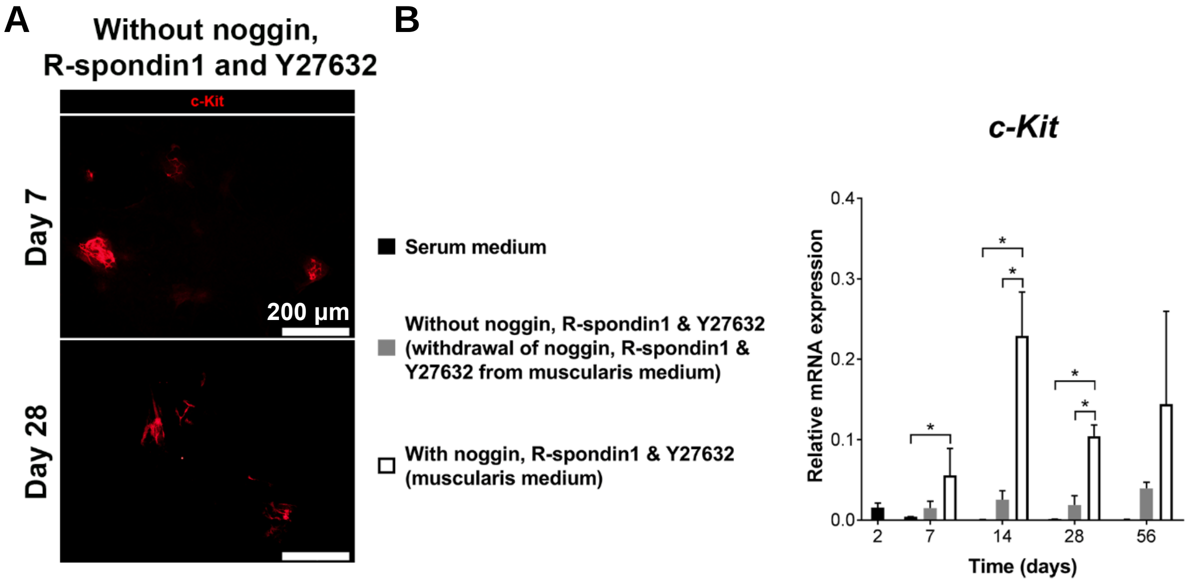

S9 Fig. The expression of c-Kit decreased when noggin, R-spondin1 and Y27632 (NRY) were removed from the muscularis medium. (**A**) Immunofluorescence of c-Kit at day 7 and 28 (n = 3 biologically independent samples). Scale bars, 200 µm. (**B**) Relative mRNA expression of *c-Kit* in the serum medium, muscularis medium and the medium without NRY at day 2 (pre-incubation in the serum medium), 7, 14, 28 and 56, measured by real-time RT-PCR. Muscle strips served as control, *Gapdh* as the housekeeping gene. Error bars, S.D. (n = 3 biologically independent samples). Experimental groups were compared by ANOVA and Tukey's post hoc method. *$p < 0.05$.
