# Supplementary material for: Bioengineered intestinal muscularis complexes with long-term spontaneous and periodic contractions
Source: PLoS One. 2018 May 2;13(5):e0195315. doi: 10.1371/journal.pone.0195315 (PMC5931477; doi:10.1371/journal.pone.0195315)
Supplement: S1 Table — (PDF) [file pone.0195315.s010.pdf]

S1 Table Antibodies, primers and probes used in the study

Primary antibodies for immunostaining

Target	Vendor	Catalog #	Dilution
Smooth muscle myosin heavy chain 11	Abcam (Cambridge, MA)	ab53219	1: 100
Neuron specific beta III Tubulin	Abcam	ab78078	1: 200
GFAP	Abcam	ab7260	1: 200
Wide spectrum cytokeratin	Abcam	ab9377	1: 200
GFP	Abcam	ab13970	1: 200
CD117 (c-Kit)	eBioscience (San Diego, CA)	16-1172-82	1: 200
Villin	Santa Cruz (San Cruz, CA)	sc-28283	1: 200
Lysozyme	Santa Cruz	sc-292850	1: 200
Mucin 2	Santa Cruz	sc-7314	1: 200
Chromogranin A	Santa Cruz	sc-393941	1: 200
Ki67	Abcam	ab16667	1: 200
E-Cadherin	BD biosciences (San Jose, CA)	610182	1: 200

Secondary antibodies for immunostaining

Product name	Vendor	Catalog #	Dilution
Alexa Fluor® 594 goat anti-mouse IgG	Life Technologies	A-11032	1: 200
Alexa Fluor® 594 goat anti-rabbit IgG	Life Technologies	A-11037	1: 200*
Alexa Fluor® 488 goat anti-mouse IgG	Life Technologies	A-11029	1: 200
Alexa Fluor® 488 goat anti-rabbit IgG	Life Technologies	A-11034	1: 200*
Alexa Fluor® 488 goat anti-chicken IgY	Abcam	ab150169	1: 200
Alexa Fluor® 594 goat anti-rat IgG	Life Technologies	A-11007	1: 200

Note: * For staining of myosin heavy chain, the dilution of the secondary antibody is 1:100.

Primers and probes for real time RT-PCR

TaqMan® Gene Expression Assays

Gene symbol	Taqman Assay ID	Gene symbol	Taqman Assay ID
Gapdh	Mm99999915_g1	*Chat*	Mm01221880_m1
Myh11	Mm00443013_m1	*Calb1*	Mm00486647_m1
Acta2	MM01546133_m1	*Vip*	Mm00660234_m1
Pdgfra	Mm00440701_m1	*Th*	Mm00447557_m1
Tubb3	Mm00727586_s1	*Nos1*	Mm01208059_m1
Rbfox3	Mm01248771_m1	*Syp*	Mm00436850_m1
S100β	Mm00485897_m1	*Dlg4*	Mm00492193_m1
Gfap	Mm01253033_m1	*GAPDH*	Hs02758991_g1
Lyz1	Mm00657323_m1	*MUC2*	Hs03005103_g1
Muc2	Mm01276696_m1	*LYZ*	Hs00426232_m1
Chga	Mm00514341_m1	*CHGA*	Hs00900375_m1
Vil1	Mm00494146_m1	*VIL1*	Hs01031724_m1
Lgr5	Mm00438890_m1	*LGR5*	Hs00969422_m1

Customized primers and probes for real time RT-PCR

Gene symbol	Sequences
c-Kit	forward primer CCGTGAACTCCATGTGGCTAAAGA,<br>reverse primer GGTGCCAGCTATTGTGCTTTACCT,<br>probe [6-FAM]-TGAACCCTCAGCCTCAGCACATAGC[Tamra-Q]

MYH11	forward primer AAGCTCTGGAAGAGGGGAAG, reverse primer GCTGAAGCCTGTTCTTGGTC
C-KIT	forward primer TGACTTACGACAGGCTCGTG, reverse primer CCACTGGCAGTACAGAAGCA
TUBB3	forward primer AACGAGGCCTCTTCTCACAA, reverse primer GGCCTGAAGAGATGTCCAAA
GFAP	forward primer ACATCGAGATCGCCACCTAC, reverse primer ATCTCCACGGTCTTCACCAC
GAPDH	forward primer CAGCCTCAAGATCATCAGCA, reverse primer TGTGGTCATGAGTCCTTCCA
