# Supplementary material for: Bioengineered intestinal muscularis complexes with long-term spontaneous and periodic contractions
Source: PLoS One. 2018 May 2;13(5):e0195315. doi: 10.1371/journal.pone.0195315 (PMC5931477; doi:10.1371/journal.pone.0195315)
Supplement: S2 Table — (PDF) [file pone.0195315.s011.pdf]

S2 Table Components in the EC medium and their possible functions in IMC culture

		Component	*In vitro* effects on IMC culture	For IMC contractility	
EC medium	Muscularis medium	Advanced DMEM/F12	Nutrient provider	Required	Nutritious basal
		B27	Role in neural differentiation and growth[1]	Required	
		N2	Role in neural differentiation and growth[1]	Required	
		HEPES (*10 mM*)	pH adjuster	Required	
		GlutaMAX (*2 mM*)	Maintenance of cell culture	Required	
		Antibiotic-antimycotic	Antibiotics	Not necessary	
		N-Acetylcysteine (Nac, *1 mM*)	Prevention of neuronal apoptosis[2]; induction of apoptosis in smooth muscle cells[3]	Having controversial effects; concentration need to be carefully decided	
		Y27632 (*10 μM*)	Pathway signal (ROCK inhibitor)[4]	Together required, synergistic effect, potentially targeting ICC and smooth muscle cells	Signaling controller
		R-Spondin1 (*1 μg/ml*)	Pathway signal (Wnt pathway enhancer)[5]		
		Noggin (*100 ng/ml*)	Pathway signal (BMP pathway inhibitor)[6]		
		EGF (*50 ng/ml*)	Cell proliferation	"Inhibiting" IMC contraction	

References

1. Fattahi F, Steinbeck JA, Kriks S, Tchieu J, Zimmer B, Kishinevsky S, et al. Deriving human ENS lineages for cell therapy and drug discovery in Hirschsprung disease. Nature. Nature Publishing Group; 2016;531: 105–109. doi:10.1038/nature16951
2. Chen S, Ren Q, Zhang J, Ye Y, Zhang Z, Xu Y, et al. N-acetyl-L-cysteine protects against cadmium-induced neuronal apoptosis by inhibiting ROS-dependent activation of Akt/mTOR pathway in mouse brain Sujuan. Neuropathol Appl Neurobiol. 2014;40: 759–777. doi:10.1016/j.pestbp.2011.02.012
3. Tsai JC, Jain M, Hsieh CM, Lee WS, Yoshizumi M, Patterson C, et al. Induction of apoptosis by pyrrolidinedithiocarbamate and N-acetylcysteine in vascular smooth muscle cells. J Biol Chem. 1996;271: 3667–70. doi:10.1074/jbc.271.7.3667
4. Pfitzer G. Regulation of myosin phosphorylation in smooth muscle. J Appl Physiol. 2001;91: 497–503.
5. Barker N. Adult intestinal stem cells: critical drivers of epithelial homeostasis and regeneration. Nat Rev Mol Cell Biol. Nature Publishing Group; 2014;15: 19–33. doi:10.1038/nrm3721
6. Noah TK, Donahue B, Shroyer NF. Intestinal development and differentiation. Exp Cell Res. Elsevier Inc.; 2011;317: 2702–10. doi:10.1016/j.yexcr.2011.09.006
