# Supplementary material for: Bioengineered intestinal muscularis complexes with long-term spontaneous and periodic contractions
Source: PLoS One. 2018 May 2;13(5):e0195315. doi: 10.1371/journal.pone.0195315 (PMC5931477; doi:10.1371/journal.pone.0195315)
Supplement: S3 Table — (PDF) [file pone.0195315.s012.pdf]

S3 Table Selected results of medium component assessment for IMC culture

A

From EC medium, subtracting:	IMC contraction
B27	–
N2	–
HEPES	–
GlutaMAX	–
N-Acetylcysteine (Nac)	–
Y27632	–
R-Spondin1	–
Noggin	–
EGF	+++

B

From EC medium, subtracting:	IMC contraction
B27, EGF	++
N2, EGF	++
HEPES, EGF	++
GlutaMAX, EGF	++
Nac, EGF	++
Y27632, EGF	++
R-Spondin1, EGF	++
Noggin & EGF	++

C

From EC medium, subtracting:	IMC contraction
Y27632, R-Spondin1, EGF	++
Y27632, Noggin, EGF	++
R-Spondin1, Noggin, EGF	++
Y27632, R-Spondin1, Noggin, EGF	++

D

From DMEM, adding:	IMC contraction
B27	–
N2	–
HEPES	–
GlutaMAX	–
N-Acetylcysteine (Nac)	–
Y27632	–
R-Spondin1	–
Noggin	–

E

From DMEM, adding:	IMC contraction
B27,N2	–
B27,Nac	–
N2, Nac	–
B27,N2,Nac	–
B27, HEPES	+

Note: (**A**-**C**) Components in EC medium were subtracted either alone or in combination at a time. (**D**-**E**) Components were added in basal DMEM either alone or in combination at a time. Prior to the evaluation of contractility, IMC were cultured for 14 days in each condition. "–", no contraction after culture for 14 days; "+", visible, spontaneous contraction; "++", visible, spontaneous, periodic contraction; "+++", visible, spontaneous, periodic contractions with a higher contraction frequency.
