# Supplementary material for: Bioengineered intestinal muscularis complexes with long-term spontaneous and periodic contractions
Source: PLoS One. 2018 May 2;13(5):e0195315. doi: 10.1371/journal.pone.0195315 (PMC5931477; doi:10.1371/journal.pone.0195315)
Supplement: S1 Note — (PDF) [file pone.0195315.s013.pdf]

S1 Note The development of the muscularis medium for IMC culture

The EC medium contained 10 different supplements (**S2 Table**). We conducted a thorough literature review and summarized potential effects of each supplement on IMC culture (**S2 Table**)[1–6]. Based on previous studies and our initial results, we hypothesized that, one or more components in EC medium inhibited IMC contraction. In addition, we assumed that antibiotic-antimycotic (ABAM, the antibiotics) in EC medium had little effect on IMC contractility and did not perform any test related to this component. To identify critical components for IMC contraction, we subtracted each component at a time from EC medium and assessed the contractility of murine IMC in resultant media after 14-day culture (**S3A Table**). We observed that, upon withdrawal of EGF from EC medium, murine IMC in this new medium (the muscularis medium) started to generate consistent periodic contractions similar to those from native tissue, implying that EGF in EC medium prevented IMC contraction. Also studies have shown that EGF can induce the dedifferentiation of smooth muscle cells[7].The "inhibitory" effect of EGF on IMC contraction was further confirmed by the fact that the established contractions of IMC in muscularis medium could be abolished by the addition of EGF at the same concentration.

We separated components in the muscularis medium into two groups: the nutritious basal group, in which components provided the basic nutrients for cell differentiation and growth; and the signaling controller group containing the signal molecules that regulates different pathways (**S2 Table**). We then subtracted components in the muscularis medium one at a time to test whether the remaining components were all required for IMC contraction. We found that removing any component from the muscularis medium attenuated but not completely abolished IMC contractions (**S3B Table**). In addition, each component alone is not sufficient to initiate contractions (**S3D Table**). These results, when combined, indicated that it is the synergistic effect of all the components in the muscularis medium that promoted the spontaneous and periodic contractions with a frequency matching that of native tissue (at day 28).

To identify the minimum components required for the initiation of IMC contraction, we first investigated the effects of signal molecules and subtracted them either alone or in combination from the muscularis medium (**S3B**, **C Table**). Our results revealed that removal of all the three signal molecules together did not abolish the contraction but reduced the contraction speed (**S3C Table**). We then analyzed the components in the nutritious basal group with emphasis on B27, N2 and Nac (**S3E Table**). These molecules are known to have positive effects on the growth and differentiation of ENS[1,8], which might be crucial for IMC contractility. We also noted that when HEPES, the pH adjustor, was added in system, more neurons were preserved. As summarized in **S3E Table**, B27, N2, and Nac, either alone or in combination, could not provide sufficient support for IMC to generate contraction within 14 days. However, in the presence of HEPES, B27 and HEPES could promote the contraction of IMC. In summary, the minimum essential factor cocktail to initiate IMC contraction was B27 plus HEPES, but cells in this medium contracted in an irregular manner. The inclusion of Noggin, R-spondin and Y27632 (NRY) in the muscularis medium improved the contractility of IMC to better match that of muscle strips, however, after removing

NRY from the muscularis medium, it was still sufficient to maintain the periodic contractions of IMC for over 56 days except with a broader distribution of periods that peaked around 5 seconds (i.e. cells contract in a slower manner).

References

1. Fattahi F, Steinbeck JA, Kriks S, Tchieu J, Zimmer B, Kishinevsky S, et al. Deriving human ENS lineages for cell therapy and drug discovery in Hirschsprung disease. Nature. Nature Publishing Group; 2016;531: 105–109. doi:10.1038/nature16951
2. Chen S, Ren Q, Zhang J, Ye Y, Zhang Z, Xu Y, et al. N-acetyl-L-cysteine protects against cadmium-induced neuronal apoptosis by inhibiting ROS-dependent activation of Akt/mTOR pathway in mouse brain Sujuan. Neuropathol Appl Neurobiol. 2014;40: 759–777. doi:10.1016/j.pestbp.2011.02.012
3. Tsai JC, Jain M, Hsieh CM, Lee WS, Yoshizumi M, Patterson C, et al. Induction of apoptosis by pyrrolidinedithiocarbamate and N-acetylcysteine in vascular smooth muscle cells. J Biol Chem. 1996;271: 3667–70. doi:10.1074/jbc.271.7.3667
4. Pfitzer G. Regulation of myosin phosphorylation in smooth muscle. J Appl Physiol. 2001;91: 497–503.
5. Barker N. Adult intestinal stem cells: critical drivers of epithelial homeostasis and regeneration. Nat Rev Mol Cell Biol. Nature Publishing Group; 2014;15: 19–33. doi:10.1038/nrm3721
6. Noah TK, Donahue B, Shroyer NF. Intestinal development and differentiation. Exp Cell Res. Elsevier Inc.; 2011;317: 2702–10. doi:10.1016/j.yexcr.2011.09.006
7. Sobue K, Hayashi K, Nishida W. Expressional regulation of smooth muscle cell-specific genes in association with phenotypic modulation. Mol Cell Biochem. 1999;190: 105–18.
8. Hansen MB. Neurohumoral control of gastrointestinal motility. Physiol Res. 2003;52: 1–30. doi:10.1051/rnd:19800701
