# Supplementary material for: Bioengineered intestinal muscularis complexes with long-term spontaneous and periodic contractions
Source: PLoS One. 2018 May 2;13(5):e0195315. doi: 10.1371/journal.pone.0195315 (PMC5931477; doi:10.1371/journal.pone.0195315)
Supplement: S2 Note — (PDF) [file pone.0195315.s014.pdf]

S2 Note Rendering the culture condition totally serum-free

Before transferring cells to muscularis medium, IMC were first plated in the serum medium for 2 days to allow cells to adhere and grow. To render the whole culture system serum-free, we replaced serum with bovine serum albumin. Although fewer cells adhered, periodic contractions could still be observed (**S22 Video**), indicating that serum was not essential.
