# Supplementary material for: Bioengineered intestinal muscularis complexes with long-term spontaneous and periodic contractions
Source: PLoS One. 2018 May 2;13(5):e0195315. doi: 10.1371/journal.pone.0195315 (PMC5931477; doi:10.1371/journal.pone.0195315)
Supplement: S1 Code — (PDF) [file pone.0195315.s037.pdf]

S1 Code Contraction frequency test for GFP cells.

```
function multiROI()

[filename,filepath,~] = uigetfile('*.*',  'All Files (*.*)');

video_handle = VideoReader(fullfile(filepath, filename));

reference_frame = read(video_handle, 1);
frame_num = video_handle.NumberOfFrames;

% compute an average intensity change map
average_intensity_change = zeros(video_handle.Height, video_handle.Width);
for frame_idx = 1 : frame_num
   curr_frame = read(video_handle, frame_idx);
   average_intensity_change = average_intensity_change + im2double(curr_frame(:, :, 2));
end
average_intensity_change = average_intensity_change ./ frame_num;


%figure(1), subplot(121); imshow(reference_frame);
figure(1), imagesc(average_intensity_change); axis image;

roi_num =input('Input the number of ROIs you want to select in this video:');
roi_masks = cell(roi_num, 1);
roi_images = cell(roi_num, 1);

for roi_idx = 1 : roi_num
   message = sprintf('%d out of %d ROI. Left click and hold to begin drawing.\nSimply lift the mouse
button to finish',...
      roi_idx, roi_num);
   uiwait(msgbox(message));
   hFH = imfreehand();
   roi_masks{roi_idx} = hFH.createMask();
   pos = hFH.getPosition();

   % define bounding box
   x1 =  max(1, min(round(min(pos(:,2))), video_handle.Height));
   y1 =  max(1, min(round(min(pos(:,1))), video_handle.Width));
   x2 =  max(1, min(round(max(pos(:,2))), video_handle.Height));
   y2 =  max(1, min(round(max(pos(:,1))), video_handle.Width));

   roi_image_r = reference_frame(:, :, 1) .* uint8(roi_masks{roi_idx});
   roi_image_g = reference_frame(:, :, 2) .* uint8(roi_masks{roi_idx});
   roi_image_b = reference_frame(:, :, 3) .* uint8(roi_masks{roi_idx});
   roi_images{roi_idx} = cat(3, ...
      roi_image_r(x1:x2, y1:y2), ...
      roi_image_g(x1:x2, y1:y2), ...
```

```
        roi_image_b(x1:x2, y1:y2));
end

fprintf('Start processing.\n');
average_intensity = zeros(frame_num, roi_num);
average_interval = 1.0 / video_handle.FrameRate;
for i = 1 : frame_num
    raw_frame = read(video_handle, i);
    green_channel = double(raw_frame(:, :, 2));
    % abandon red and blue channel, because mostly no data is available
    for roi_idx = 1 : roi_num
        average_intensity(i, roi_idx) = mean(green_channel(roi_masks{roi_idx}));
    end
end

% filter out the first dark period
thres = mean(average_intensity) - 3 * std(average_intensity);

valid_frame_indices = zeros(roi_num, 1);
for roi_idx = 1 : roi_num
    indices = sort(find(average_intensity(:, roi_idx) >= thres(roi_idx)), 'ascend');
    valid_frame_indices(roi_idx) = indices(1);
end

for roi_idx = 1 : roi_num
    figure; hold on;
    title(sprintf('ROI %d', roi_idx));
    subplot(231); imagesc(roi_images{roi_idx}(:, :, 2)); axis image; title(sprintf('Image ROI %d', roi_idx));
    axis off;

    truncated_average_intensity = average_intensity(valid_frame_indices(roi_idx):end, roi_idx);
    truncated_timestamps = [valid_frame_indices(roi_idx) : frame_num] .* average_interval;

    Y = fft(truncated_average_intensity);
    n=length(Y);
    Y=Y(1:ceil(n/2));
    n=length(Y);
    mY=abs(Y);

    subplot(233); hold on;
    FREQ=(0:n-1)*(video_handle.FrameRate /(2*n));
    semilogy(FREQ, mY);
    xlabel('Frequency (Hz)');
    title('Periodogram of Depolarization');

    subplot(232); hold on;
    xlabel('Time (seconds)');
    ylabel('Intensity (percent)');
```

```
    title('Mean Intensity Over Time');

    plot(truncated_timestamps, truncated_average_intensity);

    Z=fftshift(mY);
    f0 = (-n/2:n/2-1)*.5*(video_handle.FrameRate/(length(Y)));  % 0-centered frequency range
    subplot(235); hold on;
    plot(f0,Z);
    xlabel('Frequency (Hz)');
    title('Zero-shift Periodogram of Depolarization');

    [pks,locs] = findpeaks(Z);
    [pkvals,idx] = sort(pks,'descend'); %sort to vector
    pkvals(2); %second largest value - the first is always 0 and doesn't mean anything
    index=find(Z==pkvals(2));
    mainFrequencyStr=num2str(f0(index));
    plot(f0(index),Z(index),'r.', 'MarkerSize',25);
    text(f0(index),Z(index),['Frequency = ',mainFrequencyStr, ' Hz']);
    Frequency = f0(index);
    axis([0 ceil(10*f0(index)) 0 ceil(2*pkvals(2))]);
    fprintf('ROI %d: Frequency %f Hz\n', roi_idx, f0(index));

    % I do the same thing for the inverse of the frequency, aka the period.
    period=1./FREQ;
    subplot(236); hold on;
    plot(period,mY);
    [pks2,locs2] = findpeaks(mY);
    [pkvals2,idx2] = sort(pks2,'descend'); %sort to vector
    pkvals2(1); % largest value
    index2=find(mY==pkvals2(1));
    mainPeriodStr=num2str(period(index2));
    plot(period(index2),mY(index2),'r.', 'MarkerSize',25);
    text(period(index2),mY(index2),['Period = ',mainPeriodStr, ' Seconds']);
    xlabel('Period (seconds)');
    axis([0 ceil(2*period(index2)) 0 ceil(2*pkvals2(1))]);
    fprintf('ROI %d: Period %f seconds.\n', roi_idx, period(index2));
  end
  end
```
