# Supplementary material for: Bioengineered intestinal muscularis complexes with long-term spontaneous and periodic contractions
Source: PLoS One. 2018 May 2;13(5):e0195315. doi: 10.1371/journal.pone.0195315 (PMC5931477; doi:10.1371/journal.pone.0195315)
Supplement: S3 Code — (PDF) [file pone.0195315.s039.pdf]

S3 Code Contraction frequency test for non-GFP cells.

```
function multiROI_heq()

[filename,filepath,~] = uigetfile('*.*',  'All Files (*.*)');

video_handle = VideoReader(fullfile(filepath, filename));

frame_num = video_handle.NumberOfFrames;

% compute an average intensity change map
average_intensity_change = zeros(video_handle.Height, video_handle.Width);
average_frame_intensity = zeros(frame_num, 1);
for frame_idx = 1 : frame_num
   curr_frame = rgb2gray(im2double(read(video_handle, frame_idx)));
   average_intensity_change = average_intensity_change + curr_frame;%(:, :, 2);
   average_frame_intensity(frame_idx) = mean(mean(curr_frame));
end
average_intensity_change = average_intensity_change ./ frame_num;
[sorted_intensity, sorted_idx] = sort(average_frame_intensity);
reference_index = find(sorted_intensity >= median(average_frame_intensity));
reference_index = sorted_idx(reference_index(1));

reference_frame = rgb2gray(read(video_handle, reference_index));
reference_hist = imhist(reference_frame);

%figure(1), subplot(121); imshow(reference_frame);
figure(1), imagesc(histeq(average_intensity_change)); axis image;

roi_num =input('Input the number of ROIs you want to select in this video:');
roi_masks = cell(roi_num, 1);
roi_images = cell(roi_num, 1);

for roi_idx = 1 : roi_num
   message = sprintf('%d out of %d ROI. Left click and hold to begin drawing.\nSimply lift the mouse 
button to finish',...
      roi_idx, roi_num);
   uiwait(msgbox(message));
   hFH = imfreehand();
   roi_masks{roi_idx} = hFH.createMask();
   pos = hFH.getPosition();

   % define bounding box
   x1 =  max(1, min(round(min(pos(:,2))), video_handle.Height));
   y1 =  max(1, min(round(min(pos(:,1))), video_handle.Width));
   x2 =  max(1, min(round(max(pos(:,2))), video_handle.Height));
   y2 =  max(1, min(round(max(pos(:,1))), video_handle.Width));
```

```
    roi_image = reference_frame .* uint8(roi_masks{roi_idx});
    roi_images{roi_idx} = roi_image;
end

fprintf('Start processing.\n');
average_intensity = zeros(frame_num, roi_num);
raw_intensity = zeros(frame_num, roi_num);
average_interval = 1.0 / video_handle.FrameRate;
for i = 1 : frame_num
    raw_frame = rgb2gray(read(video_handle, i));
    raw_channel = double(raw_frame);
    green_channel = double(histeq(raw_frame, reference_hist));

    for roi_idx = 1 : roi_num
        average_intensity(i, roi_idx) = mean(green_channel(roi_masks{roi_idx}));
        raw_intensity(i, roi_idx) = mean(raw_channel(roi_masks{roi_idx}));
    end
end

for roi_idx = 1 : roi_num
    figure; title(sprintf('ROI %d', roi_idx));

    % show original roi image patch
    subplot(121);
    imagesc(roi_images{roi_idx}); axis image; axis off;
    title(sprintf('Image ROI %d', roi_idx));

    truncated_average_intensity = average_intensity(:, roi_idx);
    truncated_timestamps = (1 : frame_num) .* average_interval;

    % show average intensity over time
    subplot(122); hold on;
    plot(truncated_timestamps, truncated_average_intensity); hold on;
    filtered_average_intensity = medfilt1(truncated_average_intensity, 30);
    [pks, locs] = findpeaks(filtered_average_intensity);
    locs = truncated_timestamps(locs);
    scatter(locs, pks, 'v');
    xlabel('Time (seconds)');
    ylabel('Average Intensity');
    title('Mean Intensity Over Time');


    Y = fft(truncated_average_intensity);
    n=length(Y);
    Y=Y(1:ceil(n/2));
    n=length(Y);
    mY=abs(Y);
    Z=fftshift(mY);
```

```
    f0 = (-n/2:n/2-1)*.5*(video_handle.FrameRate/(length(Y)));  % 0-centered frequency range
    [pks,locs] = findpeaks(Z);
    [pkvals,~] = sort(pks,'descend'); %sort to vector
    index=find(Z==pkvals(2));


    fprintf('ROI %d: FFT based frequency %f(Hz).\n', ...
        roi_idx, f0(index));
    fprintf('ROI %d: FFT based period %f(seconds)\n', ...
        roi_idx, 1 ./ f0(index));

end
end
```
